# Geographic distribution of vestibular schwannomas in West Scotland between 2000-2015

**DOI:** 10.1371/journal.pone.0175489

**Published:** 2017-05-11

**Authors:** Lisa Caulley, Michael Sawada, Kelsey Hinther, Ya-tung Iris Ko, John A. Crowther, Georgios Kontorinis

**Affiliations:** 1 Department of Otolaryngology – Head and Neck Surgery, University of Ottawa, Ottawa, Ontario, Canada; 2 The Ottawa Hospital, Ottawa, Ontario, Canada; 3 Laboratory for Applied Geomatics and GIS Science (LAGGISS), Department of Geography, Environment and Geomatics, University of Ottawa, Ottawa, Ontario, Canada; 4 Undergraduate Medicine Program, University of Saskatchewan, Saskatoon, Saskatchewan, Canada; 5 Department of Otolaryngology – Head and Neck Surgery, Queen Elizabeth University Hospital, Glasgow, United Kingdom; University of Missouri Columbia, UNITED STATES

## Abstract

**Background:**

Although the natural history of vestibular schwannomas (VS) has been previously studied, few studies have investigated associated epidemiological factors, primarily because of the lack of large available cohorts.

**Objective:**

The objective of this study was to perform a multi-scale geographical analysis of the period prevalence of VS in West Scotland from 2000 to 2015.

**Methods:**

Adults diagnosed with sporadic VS were identified through the National Health Services of West Scotland database and geocoded to the unit postcode. To assess whether the cohort of VS cases could be pooled into a period prevalence measure, the locations of VS cases were analyzed by sex using Cross-L and Difference-K functions. VS period prevalence was examined at two aggregate spatial scales: the postcode district and a coarser scale of NHS Health Boards. The spatial structure of period prevalence within each level of spatial aggregation was measured using univariate global and local Moran’s I. Bivariate local Moran’s I was used to examine the between-scale variability in period prevalence from the postcode district level to the NHS Health Boards levels. Prior to spatial autocorrelation analyses, the period prevalence at the postcode district was tested for stratified spatial heterogeneity within the NHS Health Boards using Wang’s *q*-Statistic.

**Results:**

A total of 512 sporadic VS were identified in a population of over 3.1 million. Between 2000 and 2015, VS period prevalence was highest within the NHS Health Boards of Greater Glasgow and Clyde, Ayrshire and Arran and the Western Isles. However, at the NHS scale, period prevalence exhibited no spatial autocorrelation globally or locally. At the district scale, Highland exhibited the most unusual local spatial autocorrelation. Bivariate local Moran’s I results indicated general stability of period prevalence across the postcode district to Health Boards scales. However, locally, some postcode districts in Greater Glasgow and Clyde, Ayrshire and Arran exhibited unusually low district to zone spatial autocorrelation in period prevalence, as did the southern parts of the Western Isles. Some unusually high period prevalence values between the postcode district to Health Board scale were found in Tayside, Forth Valley and Dumfries and Galloway.

**Conclusion:**

Geographic variability in VS in West Scotland was identified in this patient population, showing that there are areas, even remote, with unusually high or low period prevalence. This can be partially attributed to links between primary and tertiary care. Potential genetic or environmental risk factors that may contribute to geographic variation in this disease within Scotland are also a possibility but require further investigation.

## Introduction

Vestibular schwannomas (VS), also known as acoustic neuromas, are slow-growing, benign tumors that arise from the Schwann cells lining of predominantly the vestibular component of the eighth cranial nerve [1–12]. These tumors represent approximately 6% of all intracranial neoplasms, and are the commonest neoplasm of the internal auditory canal and the cerebellopontine angle [1, 2, 4, 10, 12]. The tumor presents unilaterally in the vast majority of cases, while rarely, when they present bilaterally they are the hallmark of a hereditary disease, Neurofibromatosis type 2 (NF2) [1–8]. Several genetic alterations, including mutations in the NF2 tumor suppressor gene has been connected with the pathogenesis of NF2-related and 30–70% of sporadic VS [13–17].

Most patients with VS present with unilateral sensorineural hearing loss (94%) and tinnitus (83%). The incidence of vestibular symptoms, including vertigo and unsteadiness, in such patients varies widely (17–75% of patients at the time of diagnosis) but this is probably under-rated and under-reported [1–7, 10, 11, 18].

According to the National Health Service (NHS), one to two cases per 100,000 of VS are reported annually. One in five patients present to the otology clinic with these symptoms, however, only 1 to 2% of the patients will be diagnosed with vestibular schwannoma [6, 19].

There is substantial spatial variation in the incidence of VS worldwide. Larjavaara et al. (2011) evaluated the incidence of VS in Denmark, Sweden, Finland and Norway from 1987–2007[20]. The study demonstrated variability in the incidence by country, but an overall trend of increasing incidence of VS. The etiology of this rising trend is postulated to stem from improvements in diagnostic technology over time and physician awareness [20].

Only one recent study in the United States has examined the association between geographic variables and differences the treatment strategy of VS using a cohort of 9761 VS cases over an eight-year period [21]. That study, however, utilized spatially disjunct regions for analysis and did not examine the spatial distribution or pattern of VS across the US.

Despite the significant number of publications on VS, geographic studies are uncommon. Our objective was to evaluate the geographical distribution of VS based on an extended cohort from West Scotland.

## Methods

This study was a geographic analysis of the period prevalence of cases of VS using geographic information system (GIS) technology. As this study served audit purposes, ethical approval or patients’ consent were not required. A detailed description of the statistical code utilized in the study design is available in the appendix (S1 Appendix).

### Data source

The demographic and clinical data were accrued from the NHS of West Scotland. Subjects included all individuals diagnosed with VS through the NHS of West Scotland as documented on the West of Scotland Skull Base Multidisciplinary Meeting database. Surveillance data of VS were collected from 2000 to 2015. Diagnosis of VS was determined based on diagnostic imaging; particularly magnetic resonance imaging (MRI) of the internal auditory meatus with intravenous gadolinium administration. In very few cases where MRI was contraindicated, computed tomography of the brain with intravenous contrast was utilized.

Patients diagnosed with Neurofibromatosis type 2 were excluded from our cohort, as there is a known causal genetic background.

The VS data comprised the location (latitude and longitude coordinates) of each case at the unit postcode level (private residence). There are approximately 1.75 million unit postcodes in the UK [22]. Given that 95% of our VS case unit postcodes are constrained within areas of less than 0.5 km^2^, the true location of a VS case would be within a few hundred meters of the unit postcode centroid that we use to represent the location of a VS case. We reasonably consider the pattern of VS case locations represented by the unit postcode centroids as a point pattern at the West Scotland scale. Unit postcode centroids representing VS cases were assigned to their respective postcode district (hereafter referred to as districts) or to one of ten NHS Health Boards zones (hereafter referred to as zones) that intersected the West Scotland catchment area. Some zones in the eastern portion of the study region did not completely fall within the study catchment of the West of Scotland. Thus, population estimates for the zones were based on summing the populations of the districts that they contained. Within the zones, there were 312 postcode districts (hereafter referred to as districts). The zone and district represent two levels of spatial aggregation at which we explore the spatial structure of VS and assess the stability of period prevalence between the two spatial scales. The total population West Scotland as well as the population of each geographical area were derived from the latest national census data available by postcode [23].

### Data analysis

Mapping and data preparation were conducted with ArcGIS desktop 10.4.1 [24]. ArcGIS was used specifically to: 1) Count the number of VS cases (unit postcode centroids) within the zones and within each district using spatial joins; 2) Calculate period prevalence at both the district and zone aggregate levels; 3) Assign VS period prevalence from the zones to their nested districts using a centroid-based spatial join for purposes of exploring across scale spatial autocorrelation and stratified spatial heterogeneity; 5) produce cartographic representations of the spatial variation in VS. Spatial analysis packages (detailed below) and functions within the R v3.3.1 language were used to analyze the spatial dependency of VS nationally and across spatial scales, between the district and zone (district-zone) [25]. Stratified spatial heterogeneity between the district and zone (district-zone) level was tested using Wang’s *q*-Statistic in GeoDetector [26, 27].

#### Sex dependency

Before analyzing spatial dependency in the VS period prevalence, we needed to determine whether male and female VS case locations could be reasonably pooled into a single VS dataset. We tested for spatial interaction between male and female VS case locations using bivariate point pattern analyses based on Ripley’s K [28]. The bivariate point pattern analyses used the unit postcode VS case coordinates within a Cross-L function to test for spatial independence between the locations of male and female VS cases (S1 Appendix). This was followed by a Difference-K (K_male_-K_female_) test to detect any sex-based conditional clustering or dispersion in West Scotland (S1 Appendix). Both the Cross-L and Difference-K analyses used the R language with the Spatstat package v1.47–0 (S1 Appendix)[29]. For the Cross-L, our interest was in determining if VS cases for males and females, taken separately, exhibit significant attraction and so a one-tailed pseudo-*p* value was produced. For the Difference-K analysis, our interest was whether either of the patterns of male and female VS cases exhibited conditional independence and so a two-sided pseudo-*p* was calculated. We used 199 Monte-Carlo simulations for significance testing of each measure. Because the Monte-Carlo simulations were conditional on the same geospatial polygon layer and number of points, and there was no need to compare results with other regions, corrections for edge effects in the point pattern analyses were deemed unnecessary. Results were represented graphically as pointwise simulation envelopes to illustrate the possible outcomes of our hypotheses tests for spatial interaction at any given prespecified distance [25, 29, 30]. Statistical significance of the Cross-L and Difference-K functions across the distance interval of function evaluation (0 to 80 km at 160 m intervals) were determined via a Diggle-Cressie-Loosmore-Ford (DCLF) test (S1 Appendix) [30–32]. All K-function based analyses used Euclidean distance in a UTM Zone 30 N coordinate system.

#### Period prevalence and standardized period prevalence

The period prevalence (PP) was calculated as the number of VS cases over the 15-year period, divided by the population for each aggregated spatial unit (district and zone) [33]. All period prevalence measures were carried out assuming a national Scottish population of 3.6 million (3.16 million within the ten zones) in 2011 based on the latest census data available.

#### Stratified spatial heterogeneity—Wang’s q-Statistic

To calculate Wang’s q-Statistic, district level PP values were paired with integer codes from 1 to 10 according to their containing zone using a spatial join operation in ArcGIS. The data was exported to GeoDetector and analyzed in MS Excel.

#### Spatial autocorrelation-Moran’s I

**Fig 1:**
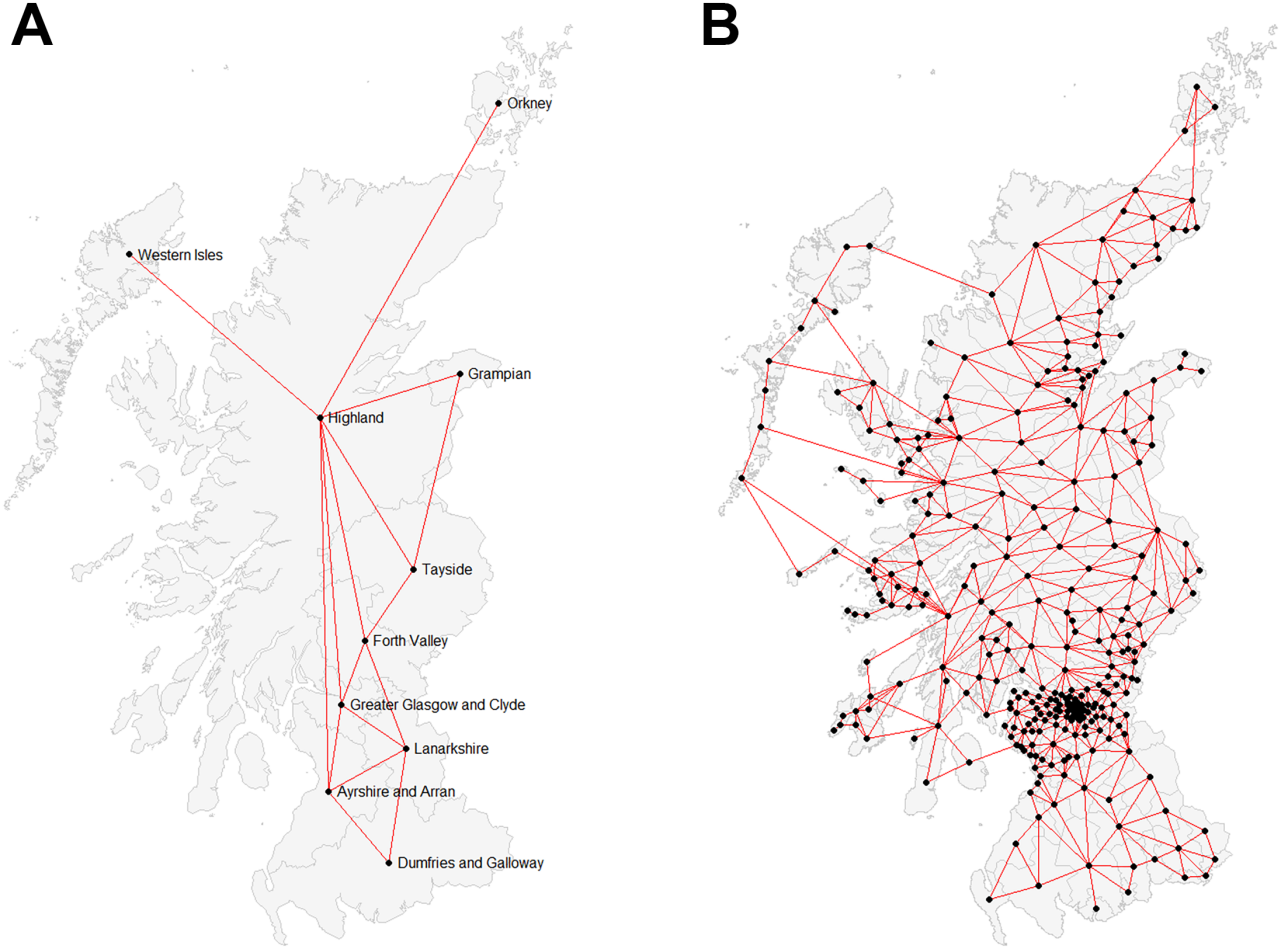
Neighbor graphs illustrating the connectivity used at the zone (a) and district level (b) for modelling spatial interaction within the spatial weights matrices used in calculating spatial autocorrelation measures.

Our model of spatial dependence for the districts and zones was based on spatial interaction. We defined spatial interaction as ferry connections between islands and the mainland or between parts of the mainland in addition to first-order Queen’s case contiguity for mainland and/or island spatial units (Fig 1a and 1b) [34].

Our definition of interaction is a reasonable basis for exploring spatial autocorrelation in VS PP. However, given the current lack of knowledge regarding the spatial processes governing VS in West Scotland, and recognizing the sensitivity of spatial autocorrelation measures to definitions of spatial dependency, we also computed all spatial autocorrelation measures using an alternate model of spatial dependence based on a k = 4 nearest neighbor adjacency matrix (S1 Appendix). Both spatial dependence schemes were row-standardized for use in spatial autocorrelation tests. As the processes governing the spatial distribution of VS in Scotland become disambiguated, more appropriate models of spatial dependency that account for spatial interaction along different dimensions could be formulated and these may produce different results [35].

Global Moran’s I was used to test spatial autocorrelation in PP at both levels of spatial aggregation (zone and district). The statistical significance of global Moran’s I was computed using 999 permutations of PP values across all spatial units (S1 Appendix). Our primary interest was detecting if global positive spatial autocorrelation exists in PP at each level of spatial aggregation and so we calculated one-sided pseudo *p*-values. All calculations of global Moran’s I were undertaken using the spdep 0.6–8 package in R[36, 37].

Functions were written in R to calculate univariate and bivariate local Moran’s I (S1 Appendix). Univariate local Moran’s I functions were validated against output from the PySAL python library and the bivariate measure was validated against the output from Geoda 1.4.6 [38, 39]. We used the R language for these calculations because of the flexibility it offered for simulations and modification of spatial neighbor matrices. For these local measures, our primary interest was to identify spatial units that exhibit unusual differences of PP values from their neighbors. A spatial unit with significant differences from neighboring values is called a cluster center. A cluster center exhibits positive local spatial autocorrelation when PP values surrounding the spatial unit are similar, for example, either a high value of PP surrounded by high values of PP (high-high) or a low value surrounded by low values (low-low). A cluster center exhibits negative local spatial autocorrelation when PP values surrounding the spatial unit are dissimilar, for example, a low PP value surrounded by high PP values (low-high) or, conversely, a high value surrounded by low values (high-low). When there is no systematic relation between the PP value within a district and its neighbors, then there is no local spatial autocorrelation present. In the univariate case, the value of local Moran’s I, at the cluster center, represents the correlation between PP and itself within the surrounding spatial units at the same spatial scale—either district PP with district PP, or zone PP with zone PP. In the bivariate case, the value of local Moran’s I at the cluster center represents the correlation between PP at the district scale to those PP values assigned to the surrounding districts from the zone level (district-zone). As such, the bivariate measure illustrates if a given district exhibits stability in PP across spatial scales. The statistical significance of the local measures were based on 9999 conditional permutations to derive the local pseudo-*p* values (S1 Appendix) [38, 40, 41]. We present both univariate and bivariate local Moran’s I results at the standard Type I error rate of α = 0.05 based on the pseudo-*p* values derived by conditional permutation, however, we did not correct for multiple testing. Therefore, a locally significant result reported herein should be considered an unusual occurrence but not necessarily a statistically significant result. Hence, local Moran’s I analyses are used in an exploratory manner, the aim of which is to identify potential districts with outlying values of PP either at the district level or between the district and zone. Alternatively, the univariate and bivariate global Moran’s I measures are statistically valid tests.

The ten zones represented one of many possible geographic zonations of the district postal geography for West Scotland. These zones are defined by the National Health Service (NHS) in Scotland. Because of the small sample size of VS cases, zone-level PP will have the most stable estimates and these zones provide meaningful boundaries from administrative perspective. Conversely, as with most administrative boundaries, the zone boundaries have no *a priori* relation to the occurrence of VS. As such, inferences based on the results of using the zones may suffer from analytical biases induced by the modifiable areal unit problem (MAUP) [42]. Specifically, the statistical significance or lack thereof, of spatial dependency in VS PP calculated using global Moran’s I could simply be due to the location of the boundaries and heterogenous area distribution among the ten zones. This is collectively known as the scale and zoning effect of the MAUP. To assess the influence of the combined scale and zoning effect of the MAUP on the results of spatial autocorrelation in PP at the zone scale, 200 random aggregations of the districts were created. A random aggregation was created by 1) randomly selecting ten postcode district polygon centroids; 2) creating a Voronoi tessellation using those ten centroids; 3) assigning the ten Voronoi polygon identifiers to the 312 district polygons; 4) aggregating the district polygons by the Voronoi polygon identifiers to create ten new randomly aggregated zones and at the same time, summing the population and number of VS cases in the random zones and calculating PP (S1 Appendix). For each random zonation, global Moran’s I was calculated and pseudo-*p* values were derived 999 Monte-Carlo simulations. By comparing our observed pseudo-p value with the reference distribution created through this process of random zonation, we can better understand how the MAUP influences the likelihood that our observed spatial autocorrelation result and interpretation would have occurred due to the choice of using the NHS Health Boards geography rather than some other ten-unit zonation.

To inform our results and interpretation of PP at the zone level, we examined where district level PP deviated from the surrounding zone level PP. Accordingly, we used bivariate local Moran’s I to assess the degree of scale invariance between the PP at the district and PP at the zone level following the methods of Nelson and Brewer (2017)[42]. Using a spatial join within ArcGIS, each district PP value was paired with its corresponding PP value from within the containing zone. This process resulted in a bivariate dataset at the district scale, whereby, each district’s PP value also had the PP value for that district extracted from the zone level. The significance of bivariate local Moran’s I was based on 9999 conditional permutations of the zone VS PP values while holding the district values constant. Bivariate Moran’s I calculated between two spatial scales, in our case, allows for the identification of districts where PP is stable (scale invariant / stationary) or unstable (non-stationary) when aggregated from the district to the zone level [42]. Consequently, identifying those districts that are unusually different from their surrounding analysis zone PP values, indicates where zone level reporting and interpretations may be unduly influenced by the MAUP as a consequence of spatial aggregation.

## Results

Geographic data were available for 512 individuals diagnosed with VS from 2000 to 2015. Of these individuals, 511 met the study inclusion criteria, due to missing data in one case. Age was available for 457 of the individuals. The mean age at the time of diagnosis of VS was 57.5 years with a minimum age of 26 years and maximum age of 88 years.

### Sex dependency

**Fig 2:**
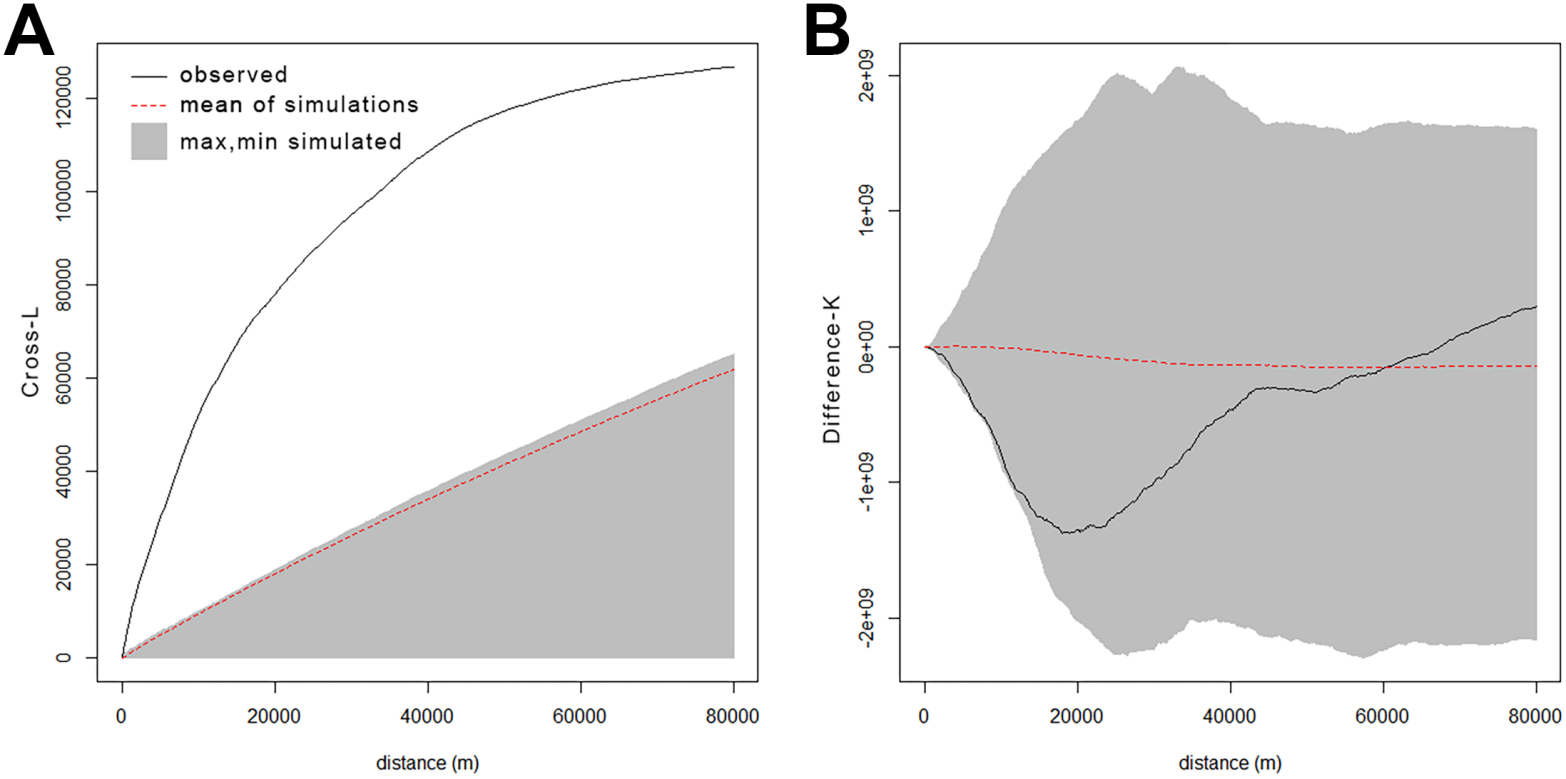
a) Cross-L function between the locations of male and female VS cases with pointwise simulation envelopes based on 199 simulations, b) Difference-K functions (male-female) with pointwise simulation envelopes from 199 random labeling of 239 male VS points.

Of the individuals diagnosed with VS in this sample population, 53.1% were female and 46.7% were male. The Cross-L results indicate that the locations of male and female VS cases exhibit spatial attraction (Fig 2a) and this is statistically significant (p < 0.005) as confirmed by a DCLF test of the Cross-L function. The Difference-K analysis results indicate that the locations of male VS cases are not conditionally dependent (and by compliment female cases) (Fig 2b) at any distance, and this is confirmed by a DCLF test (*p* = 0.34). As such, the VS cases were combined into a single point pattern for the calculation of period prevalence.

### Calculated PP and SPP

**Fig 3:**
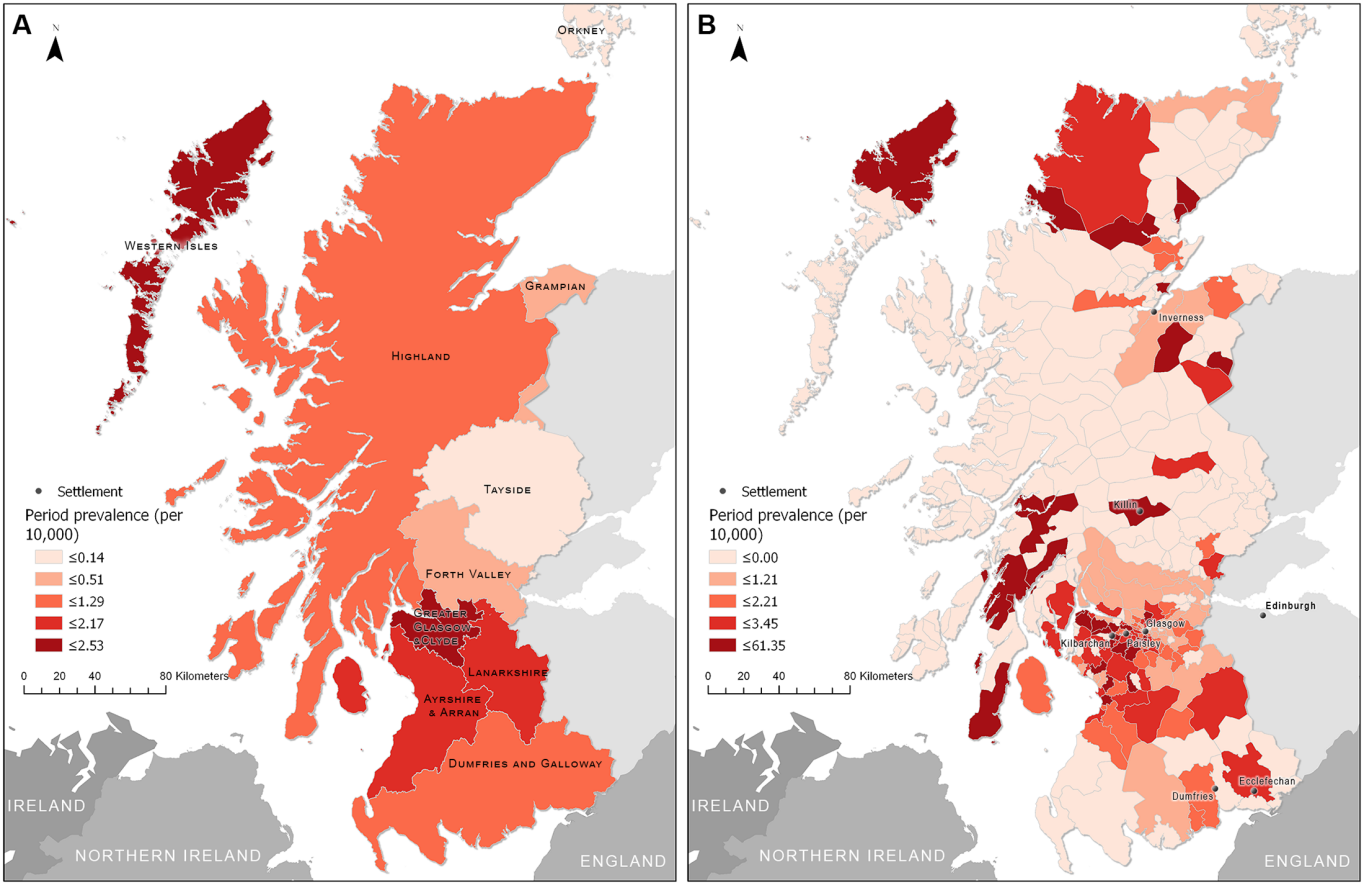
Period prevalence (SPP) of patients presenting with VS to the National Health Services of West of Scotland a) by NHS Health Boards with board names for reference and b) by postcode districts with settlements named within text. Note the different scales for period prevalence between a and b.

The aggregated geography used for PP measures is shown in Table 1. At the zone level, the Western Isles, Greater Glasgow and Clyde and Ayrshire and Arran exhibited the largest PP (Fig 3a). The lowest PP was found in Orkney, with zero VS cases reported.

**Table 1:** Period prevalence by NHS Health Boards zones.

Zone	Cases	Population	Period prevalence Cases/10 000
Ayrshire and Arran	81	373670	2.17
Dumfries and Galloway	10	151164	0.66
Forth Valley	14	275333	0.51
Grampian	2	62370	0.32
Greater Glasgow and Clyde	261	1102686	2.37
Highland	44	340903	1.29
Lanarkshire	90	666040	1.35
Orkney	0	21349	0.00
Tayside	2	142347	0.14
Western Isles	7	27684	2.53

At the district level, the highest PP is observed in the south-central area of West Scotland within the southern Inner Hebrides, in Greater Glasgow and Clyde and Ayrshire and Arron (Fig 3b). Regions near Inverness in Highland and the Isle of Harris in Western Isles also have high PP. Most districts within Highland and Tayside have the lowest PP.

### Spatial autocorrelation

PP exhibited no significant global spatial autocorrelation at the analysis zone scale using Moran’s I with our spatial dependency scheme (I = 0.125; *p* = 0.122) nor with a k = 4 neighbor definition (I = -0.131; *p* = 0.445) (S1 Appendix). From the 200 random zonations, 23% produced a significant Moran’s I using either definition of spatial dependency tested. As such, the lack of spatial dependency in VS at the zone level was not likely due to scale and zoning effects given that 77% of the random zonations showed no significant spatial autocorrelation.

At the zone scale, we observed no unusual cluster centers using Local Moran’s I (S1 Appendix).

**Fig 4:**
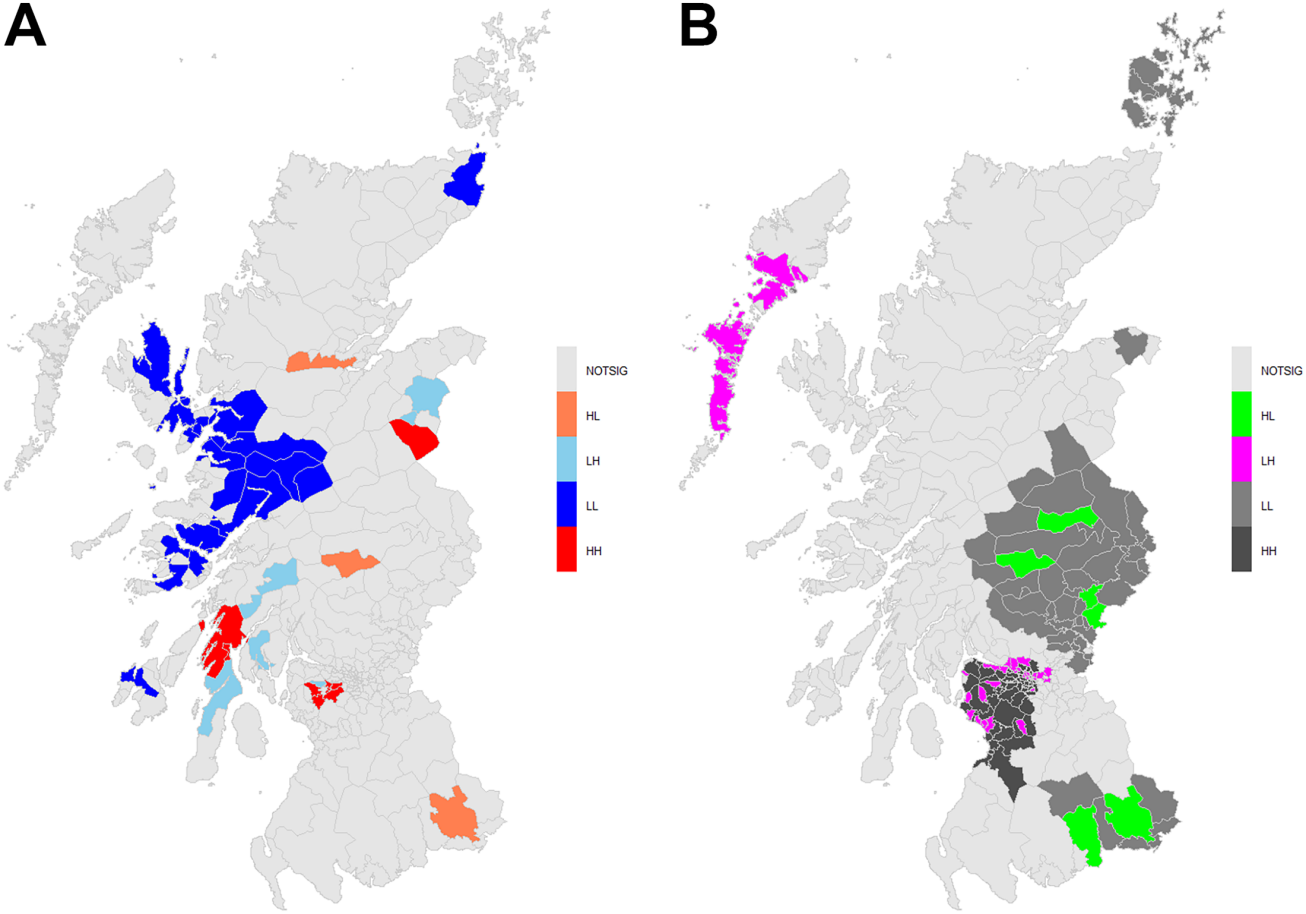
a) Univariate local Moran’s I cluster map of period prevalence (PP) at the district level; b) Bivariate local Moran’s I cluster map of period prevalence (PP) across the district-zone level. Different color schemes are used between a and b to highlight that the univariate and bivariate local Moran’s I are interpreted differently.

At the district level, using our definition of spatial dependency, global Moran’s I was not significant (I = 0.041, *p* = 0.078), nor was significance found using a k = 4 nearest neighbor spatial dependency scheme (I = 0.054047, *p* = 0.0501). However, by examining local Moran’s I results, we identified several districts with high PP surrounded by low PP (Fig 4a). These districts represented high-low cluster centers and are colored orange in Fig 4a: one centered on the town of Killin in Forth Valley, on the western shores of Loch Tay, one in Highland, north of Inverness, and one and one in the south in Dumfries and Galloway centered near Ecclefechan. At the same time, there are many contiguous low-low clusters within western Highland as shown by the blue districts in Fig 4a. There were some low-high cluster centers in the south-west and central west portions of Highland (light blue in Fig 4a). Near Paisley in Greater Glasgow and Clyde, there was a cluster of six contiguous high-high districts (red in Fig 4a). Within that cluster of high-high districts there was one low-high district whose population center is Kilbarchan in Renfrewshire.

There was significant global bivariate spatial autocorrelation (I = 0.134; *p* = 0.005) between the postcode district and the ten analysis zones. This significant positive bivariate spatial autocorrelation between the district to zone scale is expected because the same variable, PP, is being compared across two scales of spatial aggregation [42].

Wang’s *q*-statistic indicates no significant stratified spatial heterogeneity of the district PP values within the zones (*q* = 0.023; *p* = 0.652). However, bivariate local Moran’s I results at the district scale indicate areas of non-stationarity in the across-scale relation of PP (Fig 4b). For example, the grey districts in Fig 4b indicate where district level PP values are similar to neighboring zone level PP values. These cluster centers represent district-zone across-scale stability in PP values and are evident in Tayside, Forth Valley and in parts of eastern Dumfries and Galloway where low values at the district were similar to low values within adjacent districts (light grey in Fig 4b) but at the zone scale. Conversely, in Greater Glasgow and Clyde as well as Ayrshire and Arran, contained mostly high-high cluster centers (dark grey in Fig 4b) but also a large number of low-high cluster centers (magena in Fig 4b). The locations of the low-high cluster centers within Greater Glasgow and Clyde as well as Ayrshire and Arran are suggestive of districts with unusually lower values of PP than found at the containing zone level scale. Moreover, within Western Isles, south Harris, North and South Uist contained low-high cluster centers. In Tayside and Dumfries and Galloway there are a number of distinct high-low cluster centers (green in Fig 4b) where district level PP is unusually high with respect to the zone level PP value. The bivariate local Moran’s I results identify where, within the zones, there are PP deviations at the district level and hence identify where aggregation from district to zone can mask important within-zone variability.

## Discussion

### Key findings

This study provided an exploratory spatial analysis of the pattern of VS at two levels of spatial aggregation in West Scotland. This is the first study to evaluate the national geographic distribution of VS in the United Kingdom. Geographic information of this nature has the capacity to identify high-needs areas and influence resource allocation and supplementation in health policy reform.

The DCLF test of the Cross-L function results indicate that the locations of male/female cases of VS exhibited significant attraction and the DCLF test of the Difference-K function indicated that either male or female cases could be considered a random subset of the combined pattern of VS. Therefore, male and female cases co-occur and there was no significant conditional dependence of VS by sex in the West Scotland and so period prevalence could be analyzed as an aggregate of male and female cases in this study.

Within the NHS Health Boards, the highest period prevalence of VS was observed in the Western Isles, followed by the Greater Glasgow and Clyde and Ayrshire and Arran. For VS period prevalence, most of the time, ten random zonations could not detect significant spatial autocorrelation in PP within the NHS Health Boards in West Scotland. The results of the random zonation analysis suggest that MAUP scale and zoning effects have negligible impacts on our finding of a lack of global spatial structure in PP at the NHS Health Boards scale, despite different definitions of spatial dependency. Whether the lack of significant self-similarity is due to the small sample size of VS cases, or whether it is due to NHS administration at the Boards level, remains to be explored. However, it would be interesting to see if other health outcomes with similar sample sizes exhibit significant spatial autocorrelation. Moreover, there was no significant stratified spatial heterogeneity at the West Scotland scale for postcode districts within the zones. However, such heterogeneity could emerge with a different zonation at the district scale and potentially lead to aggregation induced biases as a consequence of the MAUP and this is a subject for further research.

Due to the lack of significant global spatial autocorrelation, univariate and bivariate local spatial autocorrelation were used to explore VS PP at the district scale and identify locally unusual values as well as unusual values between the district-zone scales. Through the univariate local Moran’s I, we identified several locally unusual cluster centers that exhibited low-high, high-high, low-low and high-low PP values when compared to neighboring districts. We found all cluster types in Highland; a large set of low-low cluster centers can be seen surrounding the Inner Hebrides as well as several low-high cluster centers in the south west. Bivariate local Moran’s I analysis revealed that there were pockets of local non-stationarity between PP values at the district to zone scales in Highland, Western Isles, Tayside, Forth Valley, Greater Glasgow and Clyde as well as Ayrshire and Arran. These district level deviations represent local instability that is masked within prevalence calculated within the larger scale NHS Health Boards. Such information on local deviations in prevalence at smaller spatial scales could be used to target studies on the spatial determinants of VS. Moreover, local deviations from zonal PP rates could be used in informed decisions involving health service resource allocation or programme delivery within a given NHS Health Board. For example, within larger administrative health regions like NHS Health Boards, highlighting small areas within the administrative zones that deviate from larger-scale reported rates can aid in developing knowledge-based targeted interventions or even in examining potential weak (for low-high cluster centers) or strong (for high-low cluster centers) linkages between primary and tertiary care. Scale induced biases of the MAUP are less evident within the PP measures in the central regions of West Scotland where there is less non-stationarity. However, the results of the bivariate analyses indicate that low PP values from district-zone are stable across scales in the majority of Tayside and Forth Valley, whereas high values are stable in many parts of Greater Glasgow and Clyde as well as Ayrshire and Arran.

Several factors may contribute to the geographic variation of VS seen within West Scotland including both genetic and environmental risk factors as well as the links between primary and tertiary care.

### Relation to environmental risk factors and demographics

Recently, Berkowitz et al. (2015) demonstrated evidence of environmental risk factors associated with VS, including a 4-fold increased odds of VS in individuals with a history of environmental allergies (hay fever) and individuals working in managerial/professional occupations [43]. Additionally, several studies have demonstrated similar findings in establishing an association between VS and atopic diseases [43–46]. Interestingly, Berkowitz et al. (2015) also demonstrated an inverse relationship between tobacco use and VS diagnosis [<20 pack-years odds ratio (OR) = 0.10, 95% CI = 0.04–0.28; ≥20 pack-years OR = 0.03, 95% CI = 0.01–0.12], postulating a possible anti-estrogenic effect of tobacco that may be protective, highlighting at the same time the well-known consequences of smoking [43].

Inskip et al. (2003) examined various factors in 782 patients with brain tumours, among them 96 with VS, showing that associations with indicators of affluence and education were stronger for tumours that tend to grow more slowly and have less catastrophic effects, such as VS [47]. The results of that study demonstrated a 3-fold higher odds of VS in college educated patients and a 7-fold higher odds of VS in patient’s with household income >$75,000. It is reasonable to assume that this subgroup of patients focuses more on well-being and seeks more frequently medical help, resulting in higher diagnostic rates of such tumours.

Further studies into the patient population in Scotland are needed to determine the contribution of environmental factors to the PP of VS within Scotland and give us a better insight into the underlying pathogenesis.

### Links between primary and tertiary care

Spatial variation in period prevalence of VS can also be attributed to disease surveillance practices. Stepanidis et al. (2014) demonstrated a decrease in diagnosis of VS in remote areas of Denmark due to lower rates of reporting [48]. Better links between primary and tertiary care might well result in higher diagnostic rates of VS. Carlson et al. (2016) found that geographic location impacts both VS presentation and treatment because of variability in regional referral patterns, “provider or institutional treatment preferences, and regional availability of subspecialty expertise” [21]. On the other hand, one could argue that such variabilities should not be very common within the standardized context of NHS; therefore the increased period prevalence observed in some predominantly rural areas of Scotland could well be real. However, in theory, suboptimal links between primary and tertiary care may still exist within NHS and can provide an explanation for the low-high cluster centers within certain districts found using both univariate (districts in Highlands) and bivariate (districts in Western Isles) Moran’s I.

It is worth mentioning, though, that based on Stepanidis et al. (2014) findings, one would expect unusually low PP in rural and remote regions of Scotland. Indeed, Orkney reported no VS cases. On the contrary, the Western Isles has a similar population as Orkney but had the highest PP. Moreover, consistent with Stepanidis et al. (2014), using univariate local Moran’s I, we identified a large set of low-low cluster centers around the Outer Hebrides but, conversely, we found several isolated high-low cluster centers within a number of rural districts [48]. Certainly, in the context of the NHS, there is more going on than, perhaps, issues related to surveillance practices can explain in West Scotland. However, two of the three high-low cluster centers at the district level were within a reasonable distance from major population centers. For example, the cluster center in Dumfries and Galloway is well connected to Carlisle in the England or Dumfries in Scotland by a short drive. A similar argument can be made for the cluster center found in Highland near Inverness. This generalization is less clear for the high-low cluster in Forth Valley. The explanation of optimal or suboptimal links between primary and tertiary care does not seem to apply in all cases; the high-low PP clusters are highly likely real and might be related to, for the time being, unknown factors. That being said, from a methodological perspective, given the road connections between the high-low cluster center districts and nearby larger population centers, an alternative model of spatial dependence, based on, for example, road network connections or travel time, would likely yield different local spatial autocorrelation results. Such has been demonstrated by Matisziw et al. (2008) for US ZIP codes [35]. Conversely, the low-low clusters identified within Highland are unusual, mostly rural, and could represent issues related to links between primary and tertiary care.

Moreover, the factors described by Inskip et al. (2003), such as a higher level of education resulting in higher VS diagnostic rates, can add to the possible explanation [47]; as such, patients may wish to bypass even a suboptimal referral pathway and seek medical advice and input at an earlier stage. That behavior can result in higher pick-up rates and consequently, higher documented period prevalence.

### Strengths and weaknesses

This first study of VS geography in the West of Scotland has provided a unique perspective on the period prevalence of the condition. All data were derived from one tertiary center that is responsible for the skull base service in West Scotland, ensuring a standardized diagnostic algorithm in patient selection and documentation. However, uncovering spatial determinants of VS is more difficult because such databases only contain the location of VS cases at the time of diagnosis. Although patients may not have always lived at the location reported, this is probably a shared limitation for all geographical areas examined and not only for the ones with higher/lower PP.

The primary limitation of this study stems from the relatively small number of cases for statistical analyses. However, this study identified a significant number of cases of VS compared with the numbers in the existing literature. Given the small sample size, it was not possible to examine the variation in VS PP at finer spatial scales of aggregation than the postcode district. It is possible that variation in PP at smaller spatial scales could lead to different areas exhibiting unusually high or low values of PP as has been demonstrated elsewhere [35, 49]. Analyzing the geographic distribution of VS in West Scotland is challenging because most postcode districts have low numbers of cases and low populations which make period prevalence estimates somewhat unstable and spatial aggregation necessary for rate stabilization. Spatial aggregation does, however, introduce the modifiable areal unit problem (MAUP) as a confounding factor in the analysis and the effects of the MAUP on the robustness of the results and interpretation requires further exploration [50]. Moreover, while we tested the MAUP scale and zoning effect at the zone level, we utilized a crude measure of spatial dependency that may or may not be optimal for the processes controlling VS within West Scotland.

Although our study provides a snapshot of a dynamic condition, it enhances understanding of spatial trends in VS and helps rectify the knowledge deficit regarding period prevalence of VS in Scotland. The findings should be interpreted with caution given the limitations of the study analysis tools.

## Conclusions

This study illustrated the spatial trends in VS period prevalence in West Scotland. This is the first study to undertake a spatially explicit geographic analysis of VS in the United Kingdom showing that the distribution of VS within certain areas can be unusually high or low. Our findings demonstrated the significance of geographic analysis of the period prevalence of VS in particular locations, with potential to enhance spatial targeting of interventions and policies. Careful interpretation of the findings combined with input by the sector for Public Health can help us identify factors contributing to VS and potentially other tumours.

## Supporting information

S1 Appendix Geographic distribution of vestibular schwannomas in West Scotland between 2000–2015: Geographic analysis code. (DOCX)
