# Supplementary material for: Geographic distribution of vestibular schwannomas in West Scotland between 2000-2015
Source: PLoS One. 2017 May 11;12(5):e0175489. doi: 10.1371/journal.pone.0175489 (PMC5426599; doi:10.1371/journal.pone.0175489)
Supplement: S1 Appendix — (DOCX) [file pone.0175489.s001.docx]

# S1 Appendix

# Geographic distribution of vestibular schwannomas in West Scotland between 2000-2015: Geographic Analysis Code

# Introduction

This appendix describes the methods used within "Geographic distribution of vestibular schwannomas in West Scotland between 2000-2015: an epidemiology study" and includes all code. The data referenced is available on request and the user would need to modify path strings accordingly.

Load common libraries

library(maptools)
library(spatstat)
library(sp)
library(spdep)
library(latticeExtra)

## Warning: package 'latticeExtra' was built under R version 3.3.2

Load point data. Locations of vestibular schwannoma (VS) cases at the unit postcode level geocoded and loaded into SpatialPointsDataframe object:

pts = readShapePoints("C:/Users/laggi/OneDrive/Lisa_GIS/Revision/DATA/shapefiles/vs.shp")

Load a simplified polygon for West of Scotland topologically correct

poly=readShapePoly("C:/Users/laggi/OneDrive/Lisa_GIS/Revision/DATA/shapefiles/poly2.shp",repair=T)

Create a spatstat ppp object,

pts.p=ppp(as.vector(coordinates(pts)[,1]),as.vector(coordinates(pts)[,2]),window=as.owin(poly),marks=pts$Sex)

## Warning in ppp(as.vector(coordinates(pts)[, 1]), as.vector(coordinates(pts)
## [, : data contain duplicated points

There are duplicates, e.g., unit postcode locations with more than one VS case and so have identical coordinates. So, jitter (randomly displace) all coordinates by 5 meters (m) which is negligible at this scale and has no effect on results.

pts.pp=rjitter(pts.p,5)
# Alternate tests using only unique points do show same results.
#pts.pp=unique(pts.p)

# Point Pattern Analyses

# Purpose

To determine whether or not male and female VS cases over the 15 year period can be treated as a single data set for calculating prevalence within the West Scotland. Given the small sample size (511 cases), the ability to use the full data set will maximize, as much as possible with this data, stability of period prevalence across the study area.

# Assumptions

1. If male and female VS cases exhibit attraction in space then they significantly co-occur and if neither pattern exhibits conditional dispersion or clustering with respect to the other then, spatially, both patterns could be combined for calculating period prevalence measures in the West Scotland because both patterns would be similar across the West Scotland - neither would exhibit spatial biases with respect to the other. Results could be different if age based subsets were used but because of missing age information for 53 of the VS cases stratified analysis by age cannot be completed.
2. At the scale of Scotland, the "unit postcode" (7-digit postal code location coordinates for each VS case) is considered a multitype point pattern with point type as sex, either M or F. Note: A single small user postcode may contain up to 100 addresses, but 15 is a more typical number [1]. Most VS cases occur in dense unit postcode regions; 95% of the VS cases have another unit postcode within 250 meters. Therefore, the VS locations at the unit postcode centroid are sufficient to represent the location of individual VS cases at the scale of the West Scotland.

hist(pts@data$NEAR_DIST,main="",xlab="distance")

1. Corrections for edge effects in the analysis are not necessary because Monte-Carlo simulations are conditional within the same geospatial polygon layer and on the same number of points as in the pattern being tested and we are not comparing results of either function with any other region.

## Analysis of Bivariate Pattern of Male and Female VS cases

Spatial interaction between M and F VS cases refers to stochastic dependence between events. Stochastic dependence means that the probability of an event occurring/not occurring at a particular location is dependent on whether or not an event is already nearby. Interaction can involve events in a single pattern or within multiple patterns.

## Cross-L Analyses: Do male and female VS cases exhibit spatial attraction?

One test for spatial interaction between two event patterns assumes that the two patterns are independent of each other. Thus, the probability of an event in the first pattern occurring, for example, a male VS case, has no influence on the probability of an event, a female VS case, in the second pattern occurring. If, however, the two patterns are not independent, then there may be evidence for attraction or repulsion between the two patterns. Attraction means that an event in the first pattern *increases* the probability of an event in the second pattern occurring nearby. Alternatively, the two patterns might exhibit repulsion, whereby an event in the first pattern *decreases* the probability of an event in the second pattern occurring nearby. Therefore, regarding spatial independence between *two* event patterns, we are testing the following hypotheses (one-tailed test),

- $H_{0}$: The locations of male VS cases are spatially independent of the locations of female VS cases.
- $H_{1}$: The locations of male VS cases are not spatially independent of the locations of female VS cases and they exhibit significant attraction.

The null hypothesis is also referred to as the complete spatial randomness and independence (CSRI) hypothesis for two or more patterns [2]. The chosen test for spatial independence between two patterns is called the Cross-L function, ${\overset{ˆ}{L}}_{12}(r)$ which is a variance standardized form the the cross-K function, ${\overset{ˆ}{K}}_{12}(r)$. Note that we are using a one-tailed test because our alternative hypothesis is specifically that there is attraction (co-occurrence) between the two patterns.

### ${\overset{ˆ}{\boldsymbol{L}}}_{\boldsymbol{12}}\boldsymbol{(r)}$ function analysis

Cross-K is used here in the variance standardized form ${\overset{ˆ}{L}}_{12}(r)$ based on $\overset{ˆ}{L}(r)$ from Besag [3] implemented in the spatstat function Lcross(), because this form is recommended for global envelope tests (Ibid.),

First, set the evaluation sequence for the pointwise simulation envelopes. Our function analyses are based on distance between points and that distance ranges from 0 to 80 km at intervals of 160 m.

seqeval = seq(0,80000,80000/500)

Undertake simulation,

set.seed(456)
pts.Lenv = envelope(
 pts.pp,
 Lcross,
 nrank = 1,
 nsim = 199,
 fix.n = TRUE,
 funargs = list(r = seqeval,correction = "none"),
 alternative = "greater",
 use.theory = F,
 savepatterns=T
 )

## Generating 199 simulations of CSR with fixed number of points ...
## 1, 2, 3, 4.6.8.10.12.14.16.18.20.22.24.26.28.30.32.34.36.38.
## 40.42.44.46.48.50.52.54.56.58.60.62.64.66.68.70.72.74.76.78
## .80.82.84.86.88.90.92.94.96.98.100.102.104.106.108.110.112.114.116.
## 118.120.122.124.126.128.130.132.134.136.138.140.142.144.146.148.150.152.154.156
## .158.160.162.164.166.168.170.172.174.176.178.180.182.184.186.188.190.192.194.
## 196.198 199.
##
## Done.

plot(pts.Lenv,
 xlim = range(pts.Lenv$r),
 main = expression(paste("Female,Male VS ", hat("L")[12](r)))
 )

One cannot reject nor accept a null hypothesis ($H_{0}$: The locations of male VS cases are spatially independent of the locations of female VS cases.) by searching for breaches of the simulation envelopes except at a single pre-specified distance because of issues of multiple testing/simultaneous inference e.g., one can only reject a null hypothesis using the simulation envelope test above when testing the pattern at a prespecified specific distance, for example, for whatever reason, if we are testing at a distance of 50 km only, then if the observed ${\overset{ˆ}{L}}_{12}(r)$ breaches the envelope, the null hypothesis of independence at that distance only could be rejected statistically. One can however, explore the envelope post-test and ask what the result might have been if a different distance was chosen for the test. In an exploratory manner, in this case, if any other distance had been chosen the outcome would have been the same, the null hypothesis at any distance would have been rejected in favor of the alternative.

Statistically valid global tests that reduces the ${\overset{ˆ}{L}}_{12}(r)$ function to a single test statistic within the pre-specified distance interval exist. Specifically, the Diggle-Cressie-Loosmore-Ford (DCLF) test is used here [4]. The DCLF test provides a test statistic that allows one to assess if a given ${\overset{ˆ}{L}}_{12}(r)$ produced under the null hypotheses of independence between the two point processes (as measured by across the entire distance interval for which the function is evaluated) is likely to occur by chance. For the above result pts.Lenv envelope, we use dclf.test() using the same set of simulations under the null hypothesis and same distance interval:

pts.dclf = dclf.test(
 pts.Lenv,
 Lcross,
 funargs = list(r=seqeval,correction = "none"),
 alternative="greater",
 use.theory = F
 )

## Extracting 199 point patterns from list ...
## 1, 2, 3, 4.6.8.10.12.14.16.18.20.22.24.26.28.30.32.34.36.38.
## 40.42.44.46.48.50.52.54.56.58.60.62.64.66.68.70.72.74.76.78
## .80.82.84.86.88.90.92.94.96.98.100.102.104.106.108.110.112.114.116.
## 118.120.122.124.126.128.130.132.134.136.138.140.142.144.146.148.150.152.154.156
## .158.160.162.164.166.168.170.172.174.176.178.180.182.184.186.188.190.192.194.
## 196.198 199.
##
## Done.

pts.dclf

##
## Diggle-Cressie-Loosmore-Ford test of CSR
## Monte Carlo test based on 199 simulations with fixed number of
## points
## Summary function: L["F", "M"](r)
## Reference function: sample mean
## Alternative: greater
## Interval of distance values: [0, 80000]
## Test statistic: Integral of squared signed deviation
## Deviation = leave-one-out
##
## data: pts.pp
## u = 3.3798e+14, rank = 1, p-value = 0.005

The Diggle-Cressie-Loosmore-Ford (DCLF) test yields a $p$-value = 0.005. As such, within the Interval of distance values: [0, 80000] , the null hypothesis of independence between the two processes producing a DCLF value calculated from ${\overset{ˆ}{L}}_{12}(r)$ under $H_{0}$ of 337981921805125 is rejected.. The test is conservative when parameters are estimated from the data as herein [2].

## Difference-K analyses: Do male or female VS cases exhibit conditional spatial interaction in the form of conditional dispersion or clustering?

A second question involving interaction asks whether or not one set of events is conditionally independent of another set of events. With 'conditional' independence, we are interested in how a *subset* of events of a particular kind is distributed within a larger event pattern.

In the case of conditional independence, our null hypotheses is concerned with the independence of males or females with respect to the pattern of all VS cases (males + females). Another way of saying this is that the 'males' or 'females' are a random subset of the combined pattern of males and female VS case locations. The event locations are fixed and it is the status of any given event, e.g., status = male or female, that is of interest. A reference distribution for any measure of conditional independence can be acquired by randomly labeling a subset of the combined events as a 'M' or 'F' and computing a summary statistic and then repeating the procedure $n=199$ times and each time calculating a measure of conditional independence. Specifically, we assume that the following null hypothesis is true (two-tailed),

- $H_{0}$: The locations of females are conditionally independent of the VS pattern.
- $H_{1}$: The locations of females are not conditionally independent of the VS pattern.

The chosen test is called the difference in K values, ${\overset{ˆ}{K}}_{diff}(r)$ and can be tested by randomly labeling a subset of the events as 'male' or 'female'. In other words: are the individual $\overset{ˆ}{K}(r)$ functions (${\overset{ˆ}{K}}_{M}(r)$ or ${\overset{ˆ}{K}}_{F}(r)$) equivalent? Because $VS=male+female$, we only need to test either 'male' or 'female' because the observed ${\overset{ˆ}{K}}_{diff}(r)$ of one subset (M or F) is simply the compliment of the other. If the $\overset{ˆ}{K}(r)$ functions are the same for both the 'male' and 'VS' events then by definition the 'male' (or by compliment the 'female') event pattern is a simple random subset of the combined event distribution. As such this hypothesis is called the 'random labeling hypothesis'.

### Conditional independence using ${\overset{ˆ}{\boldsymbol{K}}}_{\boldsymbol{diff}}\boldsymbol{(r)}$

The 'spatstat' library does not have a built in function for estimating ${\overset{ˆ}{K}}_{diff}(r)$ because this function is a simple difference, the following function is modified from Baddeley et al (2016 =) [2]:

Kdif = function(X, ..., i) {
 Kidot = Kdot(X, ..., i)
 K = Kest(X, ...)
 difference = eval.fv(Kidot - K)
 return(difference)
 }

This function takes in a multitype ppp object as parameter X and a character string i = 'CaseLabel' to specify which mark in the multitype pattern is to be considered as the 'case' event identifier, in our case 'F' for female VS case. The ... is a placeholder for named arguments to send to either the Kidot() function or the Kest() function that are within this function's body.

#### Undertake simulation with kdif()

ptskdiff = envelope(
 pts.pp,
 Kdif,
 nrank = 1,
 nsim = 199,
 use.theory = F,
 funargs = list(r=seqeval,correction = "none",i='M'),
 simulate = expression(rlabel(pts.pp)),
 savepatterns = T
 )

## Generating 199 simulations by evaluating expression ...
## 1, 2, 3, 4.6.8.10.12.14.16.18.20.22.24.26.28.30.32.34.36.38.
## 40.42.44.46.48.50.52.54.56.58.60.62.64.66.68.70.72.74.76.78
## .80.82.84.86.88.90.92.94.96.98.100.102.104.106.108.110.112.114.116.
## 118.120.122.124.126.128.130.132.134.136.138.140.142.144.146.148.150.152.154.156
## .158.160.162.164.166.168.170.172.174.176.178.180.182.184.186.188.190.192.194.
## 196.198 199.
##
## Done.

plot(pts.pp,
 pch = 16,
 cols = c("red", "blue")
 )

**Locations of M and F VS cases**

plot(ptskdiff,
 legend = T,
 main = expression(paste("Case-control independent ", hat("K")[diff](r)))
 )

**Difference K for M and F VS cases**

Undertake DCLF test,

ptskdiff.dclf = dclf.test(
 ptskdiff,
 Kdif,
 funargs = list(r=seqeval,correction = "none", i = 'F'),
 alternative="two.sided",
 use.theory = F
 )

## Extracting 199 point patterns from list ...
## 1, 2, 3, 4.6.8.10.12.14.16.18.20.22.24.26.28.30.32.34.36.38.
## 40.42.44.46.48.50.52.54.56.58.60.62.64.66.68.70.72.74.76.78
## .80.82.84.86.88.90.92.94.96.98.100.102.104.106.108.110.112.114.116.
## 118.120.122.124.126.128.130.132.134.136.138.140.142.144.146.148.150.152.154.156
## .158.160.162.164.166.168.170.172.174.176.178.180.182.184.186.188.190.192.194.
## 196.198 199.
##
## Done.

ptskdiff.dclf

##
## Diggle-Cressie-Loosmore-Ford test of model simulated by
## evaluating expression
## Monte Carlo test based on 199 simulations
## Summary function: K["F" ~ dot](r) - K(r)
## Reference function: sample mean
## Alternative: two.sided
## Interval of distance values: [0, 80000]
## Test statistic: Integral of squared absolute deviation
## Deviation = leave-one-out
##
## data: pts.pp
## u = 2.6298e+22, rank = 73, p-value = 0.365

The Diggle-Cressie-Loosmore-Ford (DCLF) test yields a $p$-value = 0.365. As such, within the Interval of distance values: [0, 80000] , the null hypothesis of independence between the two processes producing a DCLF value calculated from ${\overset{ˆ}{K}}_{diff}(r)$ of 26298209975220517732842 is accepted..

Plot both results together,

par(mfrow=c(1,2))

plot(pts.Lenv,
 xlim = range(pts.Lenv$r),
 main = "",
 xlab= "distance (m)",
 legendmath=F
 )

plot(ptskdiff,
 legend = F,
 main = "",
 xlab= "distance (m)",
 legendmath=F
 )

##

## Examine if spatial independence can be analyzed by age groups

Objective: To assess if spatial interaction differs among age groups. For example, do VS cases within a certain age category (40-60 yrs) deviate in their relation to the overall distribution of VS cases in the West Scotland.

First, the age data is not complete for all VS cases. There are 54 VS cases with no age information. So examine whether the VS cases with missing age information exhibit interaction using the same procedure as was done above for Male and Female cases,

ptscase = pts[pts@data$Age == 0,]
ptscontrol = pts[pts@data$Age !=0,]

pts.case = ppp(
 as.vector(coordinates(ptscase)[, 1]),
 as.vector(coordinates(ptscase)[, 2]),
 window = as.owin(poly)
)
marks(pts.case)=factor("CASE")

pts.control = ppp(
 as.vector(coordinates(ptscontrol)[, 1]),
 as.vector(coordinates(ptscontrol)[, 2]),
 window = as.owin(poly)
)

## Warning in ppp(as.vector(coordinates(ptscontrol)[, 1]),
## as.vector(coordinates(ptscontrol)[, : data contain duplicated points

marks(pts.control)=factor("CONTROL")

pts.cc=superimpose.ppp(pts.case,pts.control)

## Warning in ppp(XY$x, XY$y, window = W, marks = marx, check = check &
## needcheck): data contain duplicated points

pts.cc=rjitter(pts.cc,5)

plot(pts.cc,
 pch = 16,
 cols = c("red", "blue")
 )

ptscc.Lenv = envelope(
 pts.cc,
 Lcross,
 nrank = 1,
 nsim = 199,
 fix.n = TRUE,
 funargs = list(r = seqeval,correction = "none"),
 alternative = "greater",
 use.theory = F,
 savepatterns=T
 )

## Generating 199 simulations of CSR with fixed number of points ...
## 1, 2, 3, 4.6.8.10.12.14.16.18.20.22.24.26.28.30.32.34.36.38.
## 40.42.44.46.48.50.52.54.56.58.60.62.64.66.68.70.72.74.76.78
## .80.82.84.86.88.90.92.94.96.98.100.102.104.106.108.110.112.114.116.
## 118.120.122.124.126.128.130.132.134.136.138.140.142.144.146.148.150.152.154.156
## .158.160.162.164.166.168.170.172.174.176.178.180.182.184.186.188.190.192.194.
## 196.198 199.
##
## Done.

ptscc.dclf = dclf.test(
 ptscc.Lenv,
 Lcross,
 funargs = list(r=seqeval,correction = "none"),
 alternative="greater",
 use.theory = F
 )

## Extracting 199 point patterns from list ...
## 1, 2, 3, 4.6.8.10.12.14.16.18.20.22.24.26.28.30.32.34.36.38.
## 40.42.44.46.48.50.52.54.56.58.60.62.64.66.68.70.72.74.76.78
## .80.82.84.86.88.90.92.94.96.98.100.102.104.106.108.110.112.114.116.
## 118.120.122.124.126.128.130.132.134.136.138.140.142.144.146.148.150.152.154.156
## .158.160.162.164.166.168.170.172.174.176.178.180.182.184.186.188.190.192.194.
## 196.198 199.
##
## Done.

ptscc.dclf

##
## Diggle-Cressie-Loosmore-Ford test of CSR
## Monte Carlo test based on 199 simulations with fixed number of
## points
## Summary function: L["CASE", "CONTROL"](r)
## Reference function: sample mean
## Alternative: greater
## Interval of distance values: [0, 80000]
## Test statistic: Integral of squared signed deviation
## Deviation = leave-one-out
##
## data: pts.cc
## u = 2.3181e+14, rank = 1, p-value = 0.005

ptscckdiff = envelope(
 pts.cc,
 Kdif,
 nrank = 1,
 nsim = 199,
 use.theory = F,
 funargs = list(r=seqeval,correction = "none",i='CASE'),
 simulate = expression(rlabel(pts.cc)),
 savepatterns = T
 )

## Generating 199 simulations by evaluating expression ...
## 1, 2, 3, 4.6.8.10.12.14.16.18.20.22.24.26.28.30.32.34.36.38.
## 40.42.44.46.48.50.52.54.56.58.60.62.64.66.68.70.72.74.76.78
## .80.82.84.86.88.90.92.94.96.98.100.102.104.106.108.110.112.114.116.
## 118.120.122.124.126.128.130.132.134.136.138.140.142.144.146.148.150.152.154.156
## .158.160.162.164.166.168.170.172.174.176.178.180.182.184.186.188.190.192.194.
## 196.198 199.
##
## Done.

ptscckdiff.dclf = dclf.test(
 ptscckdiff,
 Kdif,
 funargs = list(r=seqeval,correction = "none", i = 'CASE'),
 alternative="two.sided",
 use.theory = F
 )

## Extracting 199 point patterns from list ...
## 1, 2, 3, 4.6.8.10.12.14.16.18.20.22.24.26.28.30.32.34.36.38.
## 40.42.44.46.48.50.52.54.56.58.60.62.64.66.68.70.72.74.76.78
## .80.82.84.86.88.90.92.94.96.98.100.102.104.106.108.110.112.114.116.
## 118.120.122.124.126.128.130.132.134.136.138.140.142.144.146.148.150.152.154.156
## .158.160.162.164.166.168.170.172.174.176.178.180.182.184.186.188.190.192.194.
## 196.198 199.
##
## Done.

ptskdiff.dclf

##
## Diggle-Cressie-Loosmore-Ford test of model simulated by
## evaluating expression
## Monte Carlo test based on 199 simulations
## Summary function: K["F" ~ dot](r) - K(r)
## Reference function: sample mean
## Alternative: two.sided
## Interval of distance values: [0, 80000]
## Test statistic: Integral of squared absolute deviation
## Deviation = leave-one-out
##
## data: pts.pp
## u = 2.6298e+22, rank = 73, p-value = 0.365

plot(ptscckdiff,
 legend = T,
 main = expression(paste("Case-control independent ", hat("K")[diff](r)))
 )

The Diggle-Cressie-Loosmore-Ford (DCLF) test yields a $p$-value = 0.005. As such, within the Interval of distance values: [0, 80000] , the null hypothesis of independence between the two processes producing a DCLF value calculated from ${\overset{ˆ}{L}}_{12}(r)$ under $H_{0}$ of 231809501793610 is rejected.

The Diggle-Cressie-Loosmore-Ford (DCLF) test yields a $p$-value = 0.005. As such, within the Interval of distance values: [0, 80000] , the null hypothesis of independence between the two processes producing a DCLF value calculated from ${\overset{ˆ}{K}}_{diff}(r)$ of 5315381799738497158062242 is rejected.

Therefore, although the missing VS case locations co-occur with the control set (exhibit significant attraction), there is conditional clustering of those VS cases with missing age information, and, therefore, any analysis of spatial interaction among different age groups using only those VS cases with age data available could lead to conclusions of significant or non-significant interaction that are due to the VS cases with no age information being excluded from such analyses.

Conclusion: Spatial interaction between age groups should not be attempted.

# Spatial Autocorrelation

## Assumptions

1. Interaction between the 10 analysis zones or 312 postcode districts can be modeled using 1st order neighborhood contiguity and for the Western Isles (Harris etc..) and Orkney by ferry connections to the Scottish mainland. This concept of interaction is reasonable for a first approximation as the basis to test the self-similarity of VS prevalence.
2. Alternate models of spatial dependence could produce different results. Hence, test with a k=4 nearest neighbor definition of spatial dependence to assess sensitivity of results.

## NHS Health Boards analysis scale

Data for the 10 analysis zones that represent the 10 NHS Health Boards that intersect the West of Scotland:

# Shapefile SRS
coordsys = CRS("+init=epsg:32630")

pathtozones = "C:/Users/laggi/OneDrive/Lisa_GIS/Revision/DATA/shapefiles/"

# nhs2.shp is a simplified set of polygons for the NHS Boards based on the Douglas-Peuker algoritm and a 500 m tolerance. This is to speed up R functions.

analysis_zones.shp = paste(pathtozones, "nhs2.shp", sep = "")

# 10 analysis zones
fcpolys.sp = readShapePoly(analysis_zones.shp, proj4string = coordsys, repair =
T)

Spatial auto-correlation simultaneously examines both location and attribute information. If there is no connection between the values of a mapped variable at adjacent locations, then the data exhibits a lack of spatial auto-correlation. In other words, a lack of spatial auto-correlation should be found in a mapped pattern that does not significantly deviate from a map where each location's value $z_{i}$, was assigned randomly with equal probability to each $x,y$ location on the map. Such a map would be called a random field.

Moran's I ranges from +1, strong positive spatial auto-correlation, to $-\frac{1}{(n-1)}$, signifying a pattern with values of a variable assigned randomly to each location, and finally to -1 indicating strong negative spatial auto-correlation.

For a one-tailed test, specifically,

- $H_{0}$: There is no evidence of spatial auto-correlation among the 10 analysis zones.
- $H_{1}$: There is evidence of positive spatial auto-correlation among the 10 analysis zones.

Examine the @data slot,

head(fcpolys.sp@data)

## VSCASES HBCode HBName Sum_POP PREV InPoly_FID
## 0 81 S08000015 Ayrshire and Arran 373670 2.1676881 0
## 1 10 S08000017 Dumfries and Galloway 151164 0.6615332 1
## 2 14 S08000019 Forth Valley 275333 0.5084752 2
## 3 2 S08000020 Grampian 62370 0.3206670 3
## 4 261 S08000021 Greater Glasgow and Clyde 1102686 2.3669476 4
## 5 44 S08000022 Highland 340903 1.2906897 5
## SimPgnFlag MaxSimpTol MinSimpTol UID
## 0 0 500 500 1
## 1 0 500 500 2
## 2 0 500 500 3
## 3 0 500 500 4
## 4 0 500 500 5
## 5 0 500 500 6

Plot the period prevalence,

spplot(fcpolys.sp, c("PREV"))

To compute spatial auto-correlation, create a 'neighbor' object based on queen's case contiguity using the poly2nb() function of 'spdep',

fcpolys.nb = poly2nb(fcpolys.sp, row.names=fcpolys.sp$HBName, queen=TRUE)

The poly2nb() function creates a nb object class that contains the neighbors for each polygon in the fcpolys.sp() object. The argument queen = TRUE means that neighbors for each object are determined based on queens case contiguity, that is, any polygon sharing a border or even a single point with another polygon is considered adjacent. Look at a summary of the neighbors object to assess any unconnected regions that may indicate topological issues,

summary(fcpolys.nb)

## Neighbour list object:
## Number of regions: 10
## Number of nonzero links: 26
## Percentage nonzero weights: 26
## Average number of links: 2.6
## 2 regions with no links:
## Orkney Western Isles
## Link number distribution:
##
## 0 2 3 4
## 2 2 2 4
## 2 least connected regions:
## Dumfries and Galloway Grampian with 2 links
## 4 most connected regions:
## Forth Valley Greater Glasgow and Clyde Highland Lanarkshire with 4 links

Examine neighbor graph for issues with links.

plot(fcpolys.sp,col='whitesmoke',border='grey80')

plot.nb(fcpolys.nb,coordinates(fcpolys.sp),col=2,pch=16,add=T)

text(coordinates(fcpolys.sp)[,1],coordinates(fcpolys.sp)[,2],fcpolys.sp$HBName,pos=4)

Here, Western Isles and Orkney have no linkages with the mainland. There are three major Government ferry routes from the Western Isles (HS) to the mainland [5], one from the southern HS to Oban in Paisley (PA) and Sky in Inverness (IV) as well as three routes from northern HS (Lewis,Harris, North Uist) to IV (Ullapool and Uig) and PH Mallaig. As such, the HS was made contiguous to IV, Perth (PH) and PA zones within the adjacency matrix, since they have the most interaction with WI and are connected by ferry routes. Also, Ayrshire and Arran are connected to Highland by two ferry routes in the inner Hebrides. So the adjacency matrix was modified to include these links,

fcpolys.sp$HBName[c(6,8,10)]

## [1] Highland Orkney Western Isles
## 10 Levels: Ayrshire and Arran Dumfries and Galloway ... Western Isles

fcpolys.nb[[10]]=as.integer(6)
fcpolys.nb[[8]]=as.integer(6)
fcpolys.nb[[6]]=as.integer(sort(c(8,10,fcpolys.nb[[6]])))

# Connect Aryshire and Arron to Highlands (2 ferry routes in southern inner hebrides)
fcpolys.sp$HBName[c(1,6)]

## [1] Ayrshire and Arran Highland
## 10 Levels: Ayrshire and Arran Dumfries and Galloway ... Western Isles

fcpolys.nb[[1]]=as.integer(sort(c(6,fcpolys.nb[[1]])))
fcpolys.nb[[6]]=as.integer(sort(c(1,fcpolys.nb[[6]])))

summary(fcpolys.nb)

## Neighbour list object:
## Number of regions: 10
## Number of nonzero links: 32
## Percentage nonzero weights: 32
## Average number of links: 3.2
## Link number distribution:
##
## 1 2 3 4 7
## 2 2 1 4 1
## 2 least connected regions:
## Orkney Western Isles with 1 link
## 1 most connected region:
## Highland with 7 links

Re-examine neighbor graph to confirm addition of links.

plot(fcpolys.sp,col='whitesmoke',border='grey80')

plot.nb(fcpolys.nb,coordinates(fcpolys.sp),col=2,pch=16,add=T)

text(coordinates(fcpolys.sp)[,1],coordinates(fcpolys.sp)[,2],fcpolys.sp$HBName,pos=4)

Create row-standardized spatial weights matrix:

fcpolys.wt = nb2listw(fcpolys.nb, style = "W", zero.policy = TRUE)

### Global Moran's I analysis

Compute Moran's I using 999 simulations:

set.seed(923)
fcpolys.test = moran.mc(
 fcpolys.sp$PREV,
 fcpolys.wt,
 nsim = 999,
 alternative="greater",
 zero.policy = TRUE
 )

fcpolys.test

##
## Monte-Carlo simulation of Moran I
##
## data: fcpolys.sp$PREV
## weights: fcpolys.wt
## number of simulations + 1: 1000
##
## statistic = 0.12442, observed rank = 875, p-value = 0.125
## alternative hypothesis: greater

The Moran's I test yields a $p$-value = 0.125. As such, for the Monte-Carlo simulation of Moran I, the null hypothesis of no spatial auto-correlation for VS prevalence within the 10 analysis zones is accepted..

To assess the sensitivity of this result to the number of neighbors, we undertake the same test using a constant adjacency matrix where each analysis zone is connected to its four nearest neighbors using euclidean distance,

fcpolys.kn = knearneigh(coordinates(fcpolys.sp),k=4)

Next, the function knn2nb() creates the neighbor matrix,

fcpolys.knb = knn2nb(fcpolys.kn,row.names = fcpolys.sp$REGION)

Check neighbor summary,

summary(fcpolys.knb)

## Neighbour list object:
## Number of regions: 10
## Number of nonzero links: 40
## Percentage nonzero weights: 40
## Average number of links: 4
## Non-symmetric neighbours list
## Link number distribution:
##
## 4
## 10
## 10 least connected regions:
## 1 2 3 4 5 6 7 8 9 10 with 4 links
## 10 most connected regions:
## 1 2 3 4 5 6 7 8 9 10 with 4 links

Plot neighbor graph *(knn=4)*,

plot(fcpolys.sp,col='whitesmoke',border='grey80')

plot.nb(fcpolys.knb,coordinates(fcpolys.sp),col=2,pch=16,add=T)

text(coordinates(fcpolys.sp)[,1],coordinates(fcpolys.sp)[,2],fcpolys.sp$HBName,pos=4)

The 10 polygons all have 4 neighbors.

Convert to a spatial weights matrix,

fcpolys.kwt = nb2listw(fcpolys.knb, style = "W", zero.policy = FALSE)

Undertake global Moran's I test as in the previous example,

set.seed(923)
fcpolys.ktest = moran.mc(
 fcpolys.sp$PREV,
 fcpolys.kwt,
 nsim = 999,
 alternative="greater")

fcpolys.ktest

##
## Monte-Carlo simulation of Moran I
##
## data: fcpolys.sp$PREV
## weights: fcpolys.kwt
## number of simulations + 1: 1000
##
## statistic = -0.13077, observed rank = 555, p-value = 0.445
## alternative hypothesis: greater

The Moran's I test yields a $p$-value = 0.445. As such, for the Monte-Carlo simulation of Moran I, the null hypothesis of no spatial auto-correlation for VS prevalence within the 10 analysis zones is accepted.

### Assessing sensitivity of global Moran's I to zoning and scale at the NHS Health Boards scale

In order to assess the sensitivity of the aggregation scheme that was used to produce the 10 analysis zones to zoning and scale, 200 random zonations of the postcode districts were created by 1) randomly selecting ten postcode district polygon centroids; 2) creating a voroni tessellation using those ten centroids; 3) Assigning the voroni polygon identifiers to the postcode district polygons; 4) aggregating the postcode district polygons by the voroni polygon identifiers to create the 10 new zones, and, at the same time, summing the population and number of VS cases in the new zones and calculating PP,

nsims = 250

nvoroni = 10
coordsys = CRS("+init=epsg:32630")
joinfield = "JID"

popfield = "Sum_POP"
vsfield = "VS_COUNT"

pathtodata = "C:/Users/laggi/OneDrive/Lisa_GIS/Revision/DATA/shapefiles/"

centeroids.shp = paste(pathtodata, "centeroids.shp", sep = "")
extent.shp = paste(pathtodata, "voroni_extent.shp", sep = "")
districts.shp = paste(pathtodata, "postcode_districtspr.shp", sep = "")

# Locations of potscode district centroids
centsshp = readShapePoints(centeroids.shp, proj4string = coordsys)

# Extent polygon for centroids, within which, the voroni polygons will be created
centsextnt = readShapePoly(extent.shp, proj4string = coordsys, repair =
T)

# Convdert to spatstat ppp object
cents.pp = ppp(as.vector(coordinates(centsshp)[, 1]),
as.vector(coordinates(centsshp)[, 2]),
window = as.owin(centsextnt))

polylist = list()

set.seed(342)

for (i in 1:nsims) {
 # Create voroni polygons for 10 randomly selected centroids
 aggzones = dirichlet(cents.pp[sample(1:cents.pp$n, 10), ])

 # Convert to sp SpatialPolygons object for spatial join
 aggzones = as(aggzones, "SpatialPolygons")

 # Create SPDF with unique ids for each voroni polygon
 aggzones = SpatialPolygonsDataFrame(aggzones, data.frame(zid = 1:length(aggzones)))

 # assign SRS
 proj4string(aggzones) = coordsys

 # Spatially join voroni polygons to centroids. This assigns
 # the containing voroni polygon's identifier to each postcode
 # district centroid.
 #
 centsshp@data$dissolve = over(centsshp, aggzones)$zid

 # Open district polygon layer
 districts = readShapePoly(districts.shp, proj4string = coordsys, repair =
 T)

 # Join centroid data.frame to district data.frame using common
 # field in order to access the new voroni polygon identifiers within
 # the district layer for dissolving by voroni region.
 districts = merge(districts, centsshp, by.x = joinfield, by.y = joinfield)

 # Dissolve geometry and attributes of districts
 m1 = aggregate(districts[c(popfield, vsfield)], by = list(districts$dissolve), sum)

 # m = unionSpatialPolygons(districts,as.vector(districts$dissolve[,1]))
 #
 # m = SpatialPolygonsDataFrame(m,data.frame(zid = 1:length(m)))
 #
 # plot(aggzones)

 m1$PREV = m1[[vsfield]] / m1[[popfield]] * 10000

 polylist[[i]] = m1
 print(i)
}

##
## PLEASE NOTE: The components "delsgs" and "summary" of the
## object returned by deldir() are now DATA FRAMES rather than
## matrices (as they were prior to release 0.0-18).
## See help("deldir").
##
## PLEASE NOTE: The process that deldir() uses for determining
## duplicated points has changed from that used in version
## 0.0-9 of this package (and previously). See help("deldir").

## [1] 1
## [1] 2
## [1] 3
## [1] 4
## [1] 5
## [1] 6
## [1] 7
## [1] 8
## [1] 9
## [1] 10
## [1] 11
## [1] 12
## [1] 13
## [1] 14
## [1] 15
## [1] 16
## [1] 17
## [1] 18
## [1] 19
## [1] 20
## [1] 21
## [1] 22
## [1] 23
## [1] 24
## [1] 25
## [1] 26
## [1] 27
## [1] 28
## [1] 29
## [1] 30
## [1] 31
## [1] 32
## [1] 33
## [1] 34
## [1] 35
## [1] 36
## [1] 37
## [1] 38
## [1] 39
## [1] 40
## [1] 41
## [1] 42
## [1] 43
## [1] 44
## [1] 45
## [1] 46
## [1] 47
## [1] 48
## [1] 49
## [1] 50
## [1] 51
## [1] 52
## [1] 53
## [1] 54
## [1] 55
## [1] 56
## [1] 57
## [1] 58
## [1] 59
## [1] 60
## [1] 61
## [1] 62
## [1] 63
## [1] 64
## [1] 65
## [1] 66
## [1] 67
## [1] 68
## [1] 69
## [1] 70
## [1] 71
## [1] 72
## [1] 73
## [1] 74
## [1] 75
## [1] 76
## [1] 77
## [1] 78
## [1] 79
## [1] 80
## [1] 81
## [1] 82
## [1] 83
## [1] 84
## [1] 85
## [1] 86
## [1] 87
## [1] 88
## [1] 89
## [1] 90
## [1] 91
## [1] 92
## [1] 93
## [1] 94
## [1] 95
## [1] 96
## [1] 97
## [1] 98
## [1] 99
## [1] 100
## [1] 101
## [1] 102
## [1] 103
## [1] 104
## [1] 105
## [1] 106
## [1] 107
## [1] 108
## [1] 109
## [1] 110
## [1] 111
## [1] 112
## [1] 113
## [1] 114
## [1] 115
## [1] 116
## [1] 117
## [1] 118
## [1] 119
## [1] 120
## [1] 121
## [1] 122
## [1] 123
## [1] 124
## [1] 125
## [1] 126
## [1] 127
## [1] 128
## [1] 129
## [1] 130
## [1] 131
## [1] 132
## [1] 133
## [1] 134
## [1] 135
## [1] 136
## [1] 137
## [1] 138
## [1] 139
## [1] 140
## [1] 141
## [1] 142
## [1] 143
## [1] 144
## [1] 145
## [1] 146
## [1] 147
## [1] 148
## [1] 149
## [1] 150
## [1] 151
## [1] 152
## [1] 153
## [1] 154
## [1] 155
## [1] 156
## [1] 157
## [1] 158
## [1] 159
## [1] 160
## [1] 161
## [1] 162
## [1] 163
## [1] 164
## [1] 165
## [1] 166
## [1] 167
## [1] 168
## [1] 169
## [1] 170
## [1] 171
## [1] 172
## [1] 173
## [1] 174
## [1] 175
## [1] 176
## [1] 177
## [1] 178
## [1] 179
## [1] 180
## [1] 181
## [1] 182
## [1] 183
## [1] 184
## [1] 185
## [1] 186
## [1] 187
## [1] 188
## [1] 189
## [1] 190
## [1] 191
## [1] 192
## [1] 193
## [1] 194
## [1] 195
## [1] 196
## [1] 197
## [1] 198
## [1] 199
## [1] 200
## [1] 201
## [1] 202
## [1] 203
## [1] 204
## [1] 205
## [1] 206
## [1] 207
## [1] 208
## [1] 209
## [1] 210
## [1] 211
## [1] 212
## [1] 213
## [1] 214
## [1] 215
## [1] 216
## [1] 217
## [1] 218
## [1] 219
## [1] 220
## [1] 221
## [1] 222
## [1] 223
## [1] 224
## [1] 225
## [1] 226
## [1] 227
## [1] 228
## [1] 229
## [1] 230
## [1] 231
## [1] 232
## [1] 233
## [1] 234
## [1] 235
## [1] 236
## [1] 237
## [1] 238
## [1] 239
## [1] 240
## [1] 241
## [1] 242
## [1] 243
## [1] 244
## [1] 245
## [1] 246
## [1] 247
## [1] 248
## [1] 249
## [1] 250

#### Queen's case simulation

Plot 9 randomly selected simulated zones with neighbor graphs to check,

par(mfrow=c(3,3),mai=c(0,0,0,0))

for(i in 1:9) {
 rnd = sample(1:length(polylist),1)
 rnd
 plot(polylist[[rnd]], col = 'whitesmoke', border = 'grey80')
 nbs = poly2nb(polylist[[rnd]], queen = TRUE)

 plot.nb(
 nbs,
 coordinates(polylist[[rnd]]),
 col = 2,
 pch = 16,
 add = T
 )

}

Compute Moran's I using 999 simulations for Queen's case contiguity,

# Create an output vector for the p-values for each test
outps = vector("numeric")
for (i in 1:nsims) {
 nbr = poly2nb(polylist[[i]], queen = TRUE)
 print(card(nbr))
 W = nb2listw(nbr, style = "W", zero.policy = TRUE)
 fcpolys.test = moran.mc(
 polylist[[i]]$PREV,
 W,
 nsim = 999,
 alternative = "greater",
 zero.policy = TRUE
 )

 # Only include results for simulations with full neighborhoods
 if (sum(card(nbr) == 0) == 0) {
 outps = c(outps, fcpolys.test$p.value)
 }
}

## [1] 3 5 4 2 2 4 2 5 2 3
## [1] 5 4 5 3 4 1 2 2 4 2
## [1] 5 3 4 3 6 3 4 3 2 1
## [1] 3 4 2 5 2 4 3 5 6 4
## [1] 4 2 1 2 6 4 5 4 3 3
## [1] 3 4 3 5 6 5 1 4 0 3
## [1] 4 2 3 4 2 5 4 4 5 5
## [1] 0 4 2 3 4 2 4 5 5 3
## [1] 4 3 4 3 3 4 5 2 6 2
## [1] 1 1 1 3 5 2 4 0 3 4
## [1] 4 3 3 3 6 5 3 1 3 5
## [1] 6 2 4 3 3 4 2 4 5 3
## [1] 5 1 5 3 3 3 0 4 3 3
## [1] 2 6 4 5 2 3 2 0 2 4
## [1] 5 3 2 4 1 3 6 6 3 3
## [1] 1 4 5 7 4 3 4 3 3 4
## [1] 4 3 4 2 6 2 3 5 4 3
## [1] 3 1 3 2 3 6 2 3 1 4
## [1] 4 5 5 3 2 4 4 3 1 5
## [1] 3 4 3 3 3 3 5 2 5 3
## [1] 3 4 2 5 5 3 4 3 4 1
## [1] 4 4 1 4 5 2 4 3 5 2
## [1] 5 4 2 2 4 2 2 4 1 2
## [1] 2 5 0 1 3 4 2 3 2 2
## [1] 2 3 4 0 5 3 4 4 4 1
## [1] 2 4 4 1 4 5 2 5 2 3
## [1] 5 3 6 5 5 2 4 1 4 3
## [1] 5 4 4 4 3 2 3 5 3 1
## [1] 2 4 2 5 6 3 4 3 2 3
## [1] 3 7 2 3 4 3 3 4 4 1
## [1] 1 1 2 4 2 2 3 4 5 4
## [1] 2 3 4 4 3 1 4 5 2 4
## [1] 4 2 4 1 3 6 4 3 5 4
## [1] 4 1 2 4 2 4 4 5 5 3
## [1] 4 4 2 1 5 4 2 3 4 5
## [1] 2 4 4 4 1 5 2 2 3 3
## [1] 2 4 3 5 4 1 3 3 6 3
## [1] 3 1 1 4 3 6 4 1 3 4
## [1] 5 6 2 3 5 6 4 3 3 3
## [1] 2 4 6 1 3 2 4 3 3 4
## [1] 4 3 4 2 2 6 4 4 4 5
## [1] 2 3 5 3 4 4 2 6 3 4
## [1] 6 1 3 5 1 3 4 3 4 4
## [1] 5 4 3 4 3 2 2 5 5 5
## [1] 3 4 2 4 6 4 1 4 3 3
## [1] 2 1 2 4 2 5 4 5 2 5
## [1] 5 1 3 3 4 3 4 3 4 2
## [1] 1 3 2 3 5 4 2 5 2 3
## [1] 6 6 2 3 4 4 2 4 3 4
## [1] 3 2 6 4 3 3 5 2 4 4
## [1] 4 6 2 4 2 5 4 4 2 3
## [1] 2 1 5 1 3 4 3 4 4 5
## [1] 2 2 6 4 2 4 4 3 6 1
## [1] 3 2 4 5 4 5 5 5 5 2
## [1] 4 5 1 4 3 2 5 6 4 2
## [1] 3 4 3 1 5 3 4 5 5 1
## [1] 4 5 2 3 6 2 4 2 3 3
## [1] 5 2 1 2 1 3 2 6 4 4
## [1] 4 1 2 5 4 5 3 4 3 5
## [1] 3 3 6 3 4 2 6 3 3 3
## [1] 3 5 4 5 2 4 3 4 2 4
## [1] 1 3 4 1 3 3 6 4 5 4
## [1] 3 3 4 4 3 1 6 1 3 4
## [1] 4 1 5 4 1 1 4 3 3 4
## [1] 3 1 4 2 4 5 1 2 3 5
## [1] 2 3 2 4 4 1 1 4 2 3
## [1] 2 5 3 4 5 5 4 5 4 3
## [1] 4 5 4 3 2 2 4 2 5 3
## [1] 2 1 4 2 4 3 3 4 6 3
## [1] 4 1 3 3 1 5 2 3 5 3
## [1] 2 5 4 4 2 2 3 3 5 2
## [1] 5 4 4 4 6 5 4 2 3 5
## [1] 3 4 2 2 5 4 4 3 4 5
## [1] 6 2 2 4 6 3 3 4 6 2
## [1] 3 4 6 5 3 3 4 3 3 4
## [1] 5 3 1 4 3 3 6 5 4 2
## [1] 4 4 6 5 3 1 5 2 4 4
## [1] 5 2 3 6 4 3 2 5 2 2
## [1] 3 4 2 3 1 2 6 5 3 5
## [1] 2 2 5 7 3 4 1 4 3 3
## [1] 5 5 3 3 3 4 5 3 2 3
## [1] 5 2 3 3 3 7 3 5 2 3
## [1] 2 6 2 4 2 1 5 4 4 4
## [1] 4 1 1 4 2 3 3 6 4 2
## [1] 3 3 3 7 3 3 4 3 5 4
## [1] 5 2 4 4 4 2 2 4 2 5
## [1] 3 5 6 5 4 3 3 5 2 4
## [1] 6 4 2 3 2 2 6 3 5 5
## [1] 3 5 5 5 3 5 3 3 3 3
## [1] 3 3 2 1 3 5 4 5 5 5
## [1] 3 4 5 3 3 4 4 6 2 4
## [1] 4 2 6 3 3 5 2 3 5 1
## [1] 3 3 5 4 4 4 5 3 2 5
## [1] 3 5 2 4 2 4 1 3 5 3
## [1] 5 3 4 1 5 4 5 4 3 2
## [1] 1 0 1 2 2 6 4 4 4 4
## [1] 2 5 4 4 5 2 4 3 5 4
## [1] 4 4 3 3 3 3 6 4 2 2
## [1] 2 4 4 3 5 4 4 2 5 3
## [1] 4 4 3 4 2 3 5 2 5 2
## [1] 2 3 4 4 5 3 6 3 2 6
## [1] 2 4 2 3 4 7 5 4 3 4
## [1] 6 2 2 6 4 4 4 5 3 4
## [1] 3 7 3 5 4 4 4 2 5 3
## [1] 1 3 3 3 5 4 4 3 5 5
## [1] 3 4 3 3 2 3 3 3 5 5
## [1] 4 4 3 3 2 4 4 4 2 4
## [1] 2 3 1 4 2 3 4 5 5 5
## [1] 4 5 1 2 1 1 4 3 4 3
## [1] 2 5 3 2 3 6 2 3 4 4
## [1] 3 4 4 1 3 4 6 4 4 3
## [1] 2 4 3 4 4 5 3 2 3 2
## [1] 3 2 4 2 7 4 4 4 6 4
## [1] 1 4 2 2 2 2 1 2 2 2
## [1] 3 3 1 5 4 3 3 3 5 4
## [1] 4 5 4 3 2 4 5 3 4 2
## [1] 1 4 2 5 3 6 5 3 4 3
## [1] 4 3 4 6 1 2 3 1 4 4
## [1] 4 3 3 6 6 1 3 2 5 5
## [1] 5 2 5 3 4 1 5 1 3 3
## [1] 3 4 3 2 1 2 5 3 3 6
## [1] 4 5 3 5 4 4 0 2 5 2
## [1] 4 4 4 4 2 3 2 4 1 6
## [1] 4 6 3 2 3 4 4 2 5 3
## [1] 6 4 1 1 3 2 4 4 3 2
## [1] 3 4 3 4 5 4 2 4 5 2
## [1] 4 3 4 5 5 1 1 3 2 4
## [1] 4 5 4 3 5 3 1 2 2 5
## [1] 5 4 3 5 3 3 2 4 2 5
## [1] 3 4 1 5 5 1 3 1 3 4
## [1] 3 4 3 5 4 1 4 1 1 2
## [1] 5 5 4 3 2 1 5 5 3 3
## [1] 2 3 5 3 3 2 1 3 1 5
## [1] 5 7 6 4 3 3 6 6 4 6
## [1] 3 6 4 5 4 3 4 2 3 4
## [1] 4 4 0 4 3 3 6 3 3 2
## [1] 3 4 3 4 3 4 5 3 6 3
## [1] 2 4 3 2 4 5 2 6 4 2
## [1] 1 2 3 2 4 5 4 2 3 6
## [1] 3 5 3 2 4 3 2 2 3 5
## [1] 4 2 4 3 3 1 3 4 2 4
## [1] 3 4 4 3 5 6 4 4 1 4
## [1] 4 4 5 3 4 4 3 7 4 2
## [1] 4 2 4 4 4 2 5 3 1 3
## [1] 2 2 3 2 4 1 2 3 3 6
## [1] 2 2 4 3 2 3 5 5 4 2
## [1] 4 1 5 3 2 4 1 3 5 4
## [1] 4 2 2 4 2 5 5 4 5 3
## [1] 5 3 2 5 2 6 4 5 5 3
## [1] 6 4 4 3 5 5 4 3 3 5
## [1] 7 2 4 3 3 3 4 5 4 3
## [1] 4 4 1 3 2 3 3 3 6 5
## [1] 1 4 4 4 3 4 1 3 6 4
## [1] 4 5 5 3 2 2 3 4 5 3
## [1] 6 2 1 6 3 5 6 3 5 5
## [1] 4 3 4 5 3 2 1 3 1 4
## [1] 4 5 5 4 5 3 1 2 2 3
## [1] 4 4 4 5 3 2 1 2 6 5
## [1] 2 3 1 4 3 3 4 3 4 1
## [1] 4 4 3 3 2 3 4 1 4 4
## [1] 4 2 2 2 4 5 4 4 1 4
## [1] 5 4 2 5 5 2 4 4 3 4
## [1] 5 2 6 5 1 3 4 2 4 4
## [1] 4 4 3 5 3 5 1 4 3 2
## [1] 4 2 3 4 5 1 3 4 6 4
## [1] 4 4 2 3 4 2 5 5 5 2
## [1] 4 2 2 4 4 3 2 3 5 5
## [1] 2 4 3 3 3 3 3 5 4 6
## [1] 4 5 3 3 4 4 4 4 3 2
## [1] 3 6 3 2 3 2 2 1 5 3
## [1] 4 1 6 3 2 4 2 3 0 3
## [1] 2 3 2 4 3 3 3 5 8 3
## [1] 1 1 3 3 5 4 3 3 5 2
## [1] 4 4 3 3 4 4 2 7 3 2
## [1] 3 2 5 4 6 3 4 5 2 2
## [1] 5 5 3 1 4 4 3 3 2 4
## [1] 4 5 2 6 4 5 4 2 4 4
## [1] 3 4 4 4 3 1 2 4 4 3
## [1] 2 2 3 4 5 3 5 3 2 5
## [1] 3 4 1 4 2 2 4 4 4 4
## [1] 4 4 5 4 3 2 6 2 3 3
## [1] 4 2 4 4 4 4 2 2 5 3
## [1] 4 1 2 5 4 4 3 2 5 2
## [1] 2 3 6 3 4 5 5 4 4 4
## [1] 4 4 2 3 1 3 4 5 3 3
## [1] 5 3 3 3 5 4 2 1 4 4
## [1] 3 2 4 2 4 6 5 4 3 3
## [1] 5 4 4 5 5 4 3 4 2 4
## [1] 3 4 5 4 7 4 2 3 5 3
## [1] 5 6 4 3 2 2 3 3 1 5
## [1] 5 3 3 5 4 4 2 4 6 4
## [1] 1 3 6 4 3 2 2 3 5 3
## [1] 1 3 3 3 7 4 3 4 2 4
## [1] 3 3 4 3 4 2 4 5 1 3
## [1] 4 2 1 2 4 2 4 4 5 4
## [1] 1 1 4 2 2 2 4 4 4 6
## [1] 2 5 3 4 3 4 5 3 5 2
## [1] 5 1 5 3 4 3 5 1 3 4
## [1] 3 3 3 3 2 4 4 2 4 4
## [1] 4 2 5 3 5 5 3 5 3 3
## [1] 6 3 4 4 3 3 3 5 3 2
## [1] 5 4 3 2 5 2 4 2 4 1
## [1] 3 5 4 4 1 3 5 4 5 2
## [1] 5 3 3 2 5 3 5 4 2 2
## [1] 3 3 4 3 4 5 5 2 1 4
## [1] 5 2 4 3 3 4 3 4 1 3
## [1] 4 5 3 1 2 0 3 3 5 4
## [1] 4 4 6 5 4 3 2 3 3 2
## [1] 1 1 4 5 3 5 3 3 4 3
## [1] 3 5 4 2 3 2 5 3 3 4
## [1] 4 3 3 3 2 1 4 5 1 4
## [1] 4 6 4 7 2 4 3 5 3 4
## [1] 4 5 4 3 2 5 3 5 3 6
## [1] 4 4 4 5 4 3 4 2 4 6
## [1] 5 4 2 5 2 4 3 5 4 2
## [1] 5 5 2 4 6 2 4 5 3 4
## [1] 4 4 2 6 3 3 4 3 3 6
## [1] 4 3 5 3 3 4 4 2 6 2
## [1] 6 2 3 3 2 4 5 2 2 3
## [1] 4 4 2 3 6 2 5 4 2 4
## [1] 5 2 4 3 4 4 4 3 3 6
## [1] 4 2 4 1 4 4 5 3 5 4
## [1] 2 4 3 5 3 6 3 5 2 3
## [1] 4 2 7 2 4 3 1 1 4 4
## [1] 4 4 5 3 1 4 2 4 5 4
## [1] 6 4 4 5 3 2 3 1 5 3
## [1] 5 4 5 6 3 5 3 4 3 2
## [1] 4 2 4 3 2 5 4 5 1 4
## [1] 5 5 2 4 5 3 5 4 4 3
## [1] 4 4 4 2 4 3 3 6 4 4
## [1] 4 5 4 1 5 3 1 2 2 3
## [1] 6 3 2 4 3 2 4 3 4 5
## [1] 3 3 5 4 4 5 2 2 5 5
## [1] 7 3 3 4 4 3 4 4 3 3
## [1] 4 3 3 3 6 6 6 4 4 3
## [1] 5 5 3 4 3 4 4 4 2 2
## [1] 4 4 4 1 4 4 4 1 1 1
## [1] 2 2 5 6 4 2 5 1 3 4
## [1] 5 3 4 3 5 5 3 2 2 2
## [1] 5 3 5 2 3 4 4 3 3 6
## [1] 5 6 2 4 2 4 5 3 4 3
## [1] 5 3 3 2 3 3 4 6 6 3
## [1] 3 6 4 5 4 3 5 2 5 3
## [1] 2 4 4 6 5 2 4 3 3 5
## [1] 3 2 3 7 2 4 3 4 5 3
## [1] 1 2 5 2 3 3 2 3 1 4
## [1] 3 3 4 1 4 2 0 2 5 2
## [1] 4 2 6 1 5 3 4 2 2 3
## [1] 4 5 3 1 2 5 3 4 5 4
## [1] 2 4 3 5 4 2 6 0 4 4

nsignifs=sum(outps<=0.05)/length(outps)

nsignifs

## [1] 0.2372881

#### knn = 4 simulation

Plot 10 randomly selected simulated zones with nearest neighbor (knn=4) graphs.

par(mfrow=c(3,3),mai=c(0,0,0,0))

for(i in 1:9) {
 rnd = sample(1:length(polylist),1)
 rnd
 plot(polylist[[rnd]], col = 'whitesmoke', border = 'grey80')
 rnd.kn = knearneigh(coordinates(polylist[[rnd]]),k=4)
 nbs = knn2nb(rnd.kn)

 plot.nb(
 nbs,
 coordinates(polylist[[rnd]]),
 col = 2,
 pch = 16,
 add = T
 )

}

Compute Moran's I using 999 simulations for knn=4 case contiguity:

# Create an output vector for the p-values for each test
outpsk = vector("numeric")
for (i in 1:nsims) {
 rnd.kn = knearneigh(coordinates(polylist[[i]]),k=4)
 nbrk = knn2nb(rnd.kn)
 print(card(nbrk)) # just check all are 4
 W = nb2listw(nbrk, style = "W", zero.policy = TRUE)
 fcpolys.test = moran.mc(
 polylist[[i]]$PREV,
 W,
 nsim = 999,
 alternative = "greater",
 zero.policy = TRUE
 )

 # Only include results for simulations with full neighborhoods
 # which is all here for knn=4
 if (sum(card(nbrk) == 0) == 0) {
 outpsk = c(outps, fcpolys.test$p.value)
 }
}

## [1] 4 4 4 4 4 4 4 4 4 4
## [1] 4 4 4 4 4 4 4 4 4 4
## [1] 4 4 4 4 4 4 4 4 4 4
## [1] 4 4 4 4 4 4 4 4 4 4
## [1] 4 4 4 4 4 4 4 4 4 4
## [1] 4 4 4 4 4 4 4 4 4 4
## [1] 4 4 4 4 4 4 4 4 4 4
## [1] 4 4 4 4 4 4 4 4 4 4
## [1] 4 4 4 4 4 4 4 4 4 4
## [1] 4 4 4 4 4 4 4 4 4 4
## [1] 4 4 4 4 4 4 4 4 4 4
## [1] 4 4 4 4 4 4 4 4 4 4
## [1] 4 4 4 4 4 4 4 4 4 4
## [1] 4 4 4 4 4 4 4 4 4 4
## [1] 4 4 4 4 4 4 4 4 4 4
## [1] 4 4 4 4 4 4 4 4 4 4
## [1] 4 4 4 4 4 4 4 4 4 4
## [1] 4 4 4 4 4 4 4 4 4 4
## [1] 4 4 4 4 4 4 4 4 4 4
## [1] 4 4 4 4 4 4 4 4 4 4
## [1] 4 4 4 4 4 4 4 4 4 4
## [1] 4 4 4 4 4 4 4 4 4 4
## [1] 4 4 4 4 4 4 4 4 4 4
## [1] 4 4 4 4 4 4 4 4 4 4
## [1] 4 4 4 4 4 4 4 4 4 4
## [1] 4 4 4 4 4 4 4 4 4 4
## [1] 4 4 4 4 4 4 4 4 4 4
## [1] 4 4 4 4 4 4 4 4 4 4
## [1] 4 4 4 4 4 4 4 4 4 4
## [1] 4 4 4 4 4 4 4 4 4 4
## [1] 4 4 4 4 4 4 4 4 4 4
## [1] 4 4 4 4 4 4 4 4 4 4
## [1] 4 4 4 4 4 4 4 4 4 4
## [1] 4 4 4 4 4 4 4 4 4 4
## [1] 4 4 4 4 4 4 4 4 4 4
## [1] 4 4 4 4 4 4 4 4 4 4
## [1] 4 4 4 4 4 4 4 4 4 4
## [1] 4 4 4 4 4 4 4 4 4 4
## [1] 4 4 4 4 4 4 4 4 4 4
## [1] 4 4 4 4 4 4 4 4 4 4
## [1] 4 4 4 4 4 4 4 4 4 4
## [1] 4 4 4 4 4 4 4 4 4 4
## [1] 4 4 4 4 4 4 4 4 4 4
## [1] 4 4 4 4 4 4 4 4 4 4
## [1] 4 4 4 4 4 4 4 4 4 4
## [1] 4 4 4 4 4 4 4 4 4 4
## [1] 4 4 4 4 4 4 4 4 4 4
## [1] 4 4 4 4 4 4 4 4 4 4
## [1] 4 4 4 4 4 4 4 4 4 4
## [1] 4 4 4 4 4 4 4 4 4 4
## [1] 4 4 4 4 4 4 4 4 4 4
## [1] 4 4 4 4 4 4 4 4 4 4
## [1] 4 4 4 4 4 4 4 4 4 4
## [1] 4 4 4 4 4 4 4 4 4 4
## [1] 4 4 4 4 4 4 4 4 4 4
## [1] 4 4 4 4 4 4 4 4 4 4
## [1] 4 4 4 4 4 4 4 4 4 4
## [1] 4 4 4 4 4 4 4 4 4 4
## [1] 4 4 4 4 4 4 4 4 4 4
## [1] 4 4 4 4 4 4 4 4 4 4
## [1] 4 4 4 4 4 4 4 4 4 4
## [1] 4 4 4 4 4 4 4 4 4 4
## [1] 4 4 4 4 4 4 4 4 4 4
## [1] 4 4 4 4 4 4 4 4 4 4
## [1] 4 4 4 4 4 4 4 4 4 4
## [1] 4 4 4 4 4 4 4 4 4 4
## [1] 4 4 4 4 4 4 4 4 4 4
## [1] 4 4 4 4 4 4 4 4 4 4
## [1] 4 4 4 4 4 4 4 4 4 4
## [1] 4 4 4 4 4 4 4 4 4 4
## [1] 4 4 4 4 4 4 4 4 4 4
## [1] 4 4 4 4 4 4 4 4 4 4
## [1] 4 4 4 4 4 4 4 4 4 4
## [1] 4 4 4 4 4 4 4 4 4 4
## [1] 4 4 4 4 4 4 4 4 4 4
## [1] 4 4 4 4 4 4 4 4 4 4
## [1] 4 4 4 4 4 4 4 4 4 4
## [1] 4 4 4 4 4 4 4 4 4 4
## [1] 4 4 4 4 4 4 4 4 4 4
## [1] 4 4 4 4 4 4 4 4 4 4
## [1] 4 4 4 4 4 4 4 4 4 4
## [1] 4 4 4 4 4 4 4 4 4 4
## [1] 4 4 4 4 4 4 4 4 4 4
## [1] 4 4 4 4 4 4 4 4 4 4
## [1] 4 4 4 4 4 4 4 4 4 4
## [1] 4 4 4 4 4 4 4 4 4 4
## [1] 4 4 4 4 4 4 4 4 4 4
## [1] 4 4 4 4 4 4 4 4 4 4
## [1] 4 4 4 4 4 4 4 4 4 4
## [1] 4 4 4 4 4 4 4 4 4 4
## [1] 4 4 4 4 4 4 4 4 4 4
## [1] 4 4 4 4 4 4 4 4 4 4
## [1] 4 4 4 4 4 4 4 4 4 4
## [1] 4 4 4 4 4 4 4 4 4 4
## [1] 4 4 4 4 4 4 4 4 4 4
## [1] 4 4 4 4 4 4 4 4 4 4
## [1] 4 4 4 4 4 4 4 4 4 4
## [1] 4 4 4 4 4 4 4 4 4 4
## [1] 4 4 4 4 4 4 4 4 4 4
## [1] 4 4 4 4 4 4 4 4 4 4
## [1] 4 4 4 4 4 4 4 4 4 4
## [1] 4 4 4 4 4 4 4 4 4 4
## [1] 4 4 4 4 4 4 4 4 4 4
## [1] 4 4 4 4 4 4 4 4 4 4
## [1] 4 4 4 4 4 4 4 4 4 4
## [1] 4 4 4 4 4 4 4 4 4 4
## [1] 4 4 4 4 4 4 4 4 4 4
## [1] 4 4 4 4 4 4 4 4 4 4
## [1] 4 4 4 4 4 4 4 4 4 4
## [1] 4 4 4 4 4 4 4 4 4 4
## [1] 4 4 4 4 4 4 4 4 4 4
## [1] 4 4 4 4 4 4 4 4 4 4
## [1] 4 4 4 4 4 4 4 4 4 4
## [1] 4 4 4 4 4 4 4 4 4 4
## [1] 4 4 4 4 4 4 4 4 4 4
## [1] 4 4 4 4 4 4 4 4 4 4
## [1] 4 4 4 4 4 4 4 4 4 4
## [1] 4 4 4 4 4 4 4 4 4 4
## [1] 4 4 4 4 4 4 4 4 4 4
## [1] 4 4 4 4 4 4 4 4 4 4
## [1] 4 4 4 4 4 4 4 4 4 4
## [1] 4 4 4 4 4 4 4 4 4 4
## [1] 4 4 4 4 4 4 4 4 4 4
## [1] 4 4 4 4 4 4 4 4 4 4
## [1] 4 4 4 4 4 4 4 4 4 4
## [1] 4 4 4 4 4 4 4 4 4 4
## [1] 4 4 4 4 4 4 4 4 4 4
## [1] 4 4 4 4 4 4 4 4 4 4
## [1] 4 4 4 4 4 4 4 4 4 4
## [1] 4 4 4 4 4 4 4 4 4 4
## [1] 4 4 4 4 4 4 4 4 4 4
## [1] 4 4 4 4 4 4 4 4 4 4
## [1] 4 4 4 4 4 4 4 4 4 4
## [1] 4 4 4 4 4 4 4 4 4 4
## [1] 4 4 4 4 4 4 4 4 4 4
## [1] 4 4 4 4 4 4 4 4 4 4
## [1] 4 4 4 4 4 4 4 4 4 4
## [1] 4 4 4 4 4 4 4 4 4 4
## [1] 4 4 4 4 4 4 4 4 4 4
## [1] 4 4 4 4 4 4 4 4 4 4
## [1] 4 4 4 4 4 4 4 4 4 4
## [1] 4 4 4 4 4 4 4 4 4 4
## [1] 4 4 4 4 4 4 4 4 4 4
## [1] 4 4 4 4 4 4 4 4 4 4
## [1] 4 4 4 4 4 4 4 4 4 4
## [1] 4 4 4 4 4 4 4 4 4 4
## [1] 4 4 4 4 4 4 4 4 4 4
## [1] 4 4 4 4 4 4 4 4 4 4
## [1] 4 4 4 4 4 4 4 4 4 4
## [1] 4 4 4 4 4 4 4 4 4 4
## [1] 4 4 4 4 4 4 4 4 4 4
## [1] 4 4 4 4 4 4 4 4 4 4
## [1] 4 4 4 4 4 4 4 4 4 4
## [1] 4 4 4 4 4 4 4 4 4 4
## [1] 4 4 4 4 4 4 4 4 4 4
## [1] 4 4 4 4 4 4 4 4 4 4
## [1] 4 4 4 4 4 4 4 4 4 4
## [1] 4 4 4 4 4 4 4 4 4 4
## [1] 4 4 4 4 4 4 4 4 4 4
## [1] 4 4 4 4 4 4 4 4 4 4
## [1] 4 4 4 4 4 4 4 4 4 4
## [1] 4 4 4 4 4 4 4 4 4 4
## [1] 4 4 4 4 4 4 4 4 4 4
## [1] 4 4 4 4 4 4 4 4 4 4
## [1] 4 4 4 4 4 4 4 4 4 4
## [1] 4 4 4 4 4 4 4 4 4 4
## [1] 4 4 4 4 4 4 4 4 4 4
## [1] 4 4 4 4 4 4 4 4 4 4
## [1] 4 4 4 4 4 4 4 4 4 4
## [1] 4 4 4 4 4 4 4 4 4 4
## [1] 4 4 4 4 4 4 4 4 4 4
## [1] 4 4 4 4 4 4 4 4 4 4
## [1] 4 4 4 4 4 4 4 4 4 4
## [1] 4 4 4 4 4 4 4 4 4 4
## [1] 4 4 4 4 4 4 4 4 4 4
## [1] 4 4 4 4 4 4 4 4 4 4
## [1] 4 4 4 4 4 4 4 4 4 4
## [1] 4 4 4 4 4 4 4 4 4 4
## [1] 4 4 4 4 4 4 4 4 4 4
## [1] 4 4 4 4 4 4 4 4 4 4
## [1] 4 4 4 4 4 4 4 4 4 4
## [1] 4 4 4 4 4 4 4 4 4 4
## [1] 4 4 4 4 4 4 4 4 4 4
## [1] 4 4 4 4 4 4 4 4 4 4
## [1] 4 4 4 4 4 4 4 4 4 4
## [1] 4 4 4 4 4 4 4 4 4 4
## [1] 4 4 4 4 4 4 4 4 4 4
## [1] 4 4 4 4 4 4 4 4 4 4
## [1] 4 4 4 4 4 4 4 4 4 4
## [1] 4 4 4 4 4 4 4 4 4 4
## [1] 4 4 4 4 4 4 4 4 4 4
## [1] 4 4 4 4 4 4 4 4 4 4
## [1] 4 4 4 4 4 4 4 4 4 4
## [1] 4 4 4 4 4 4 4 4 4 4
## [1] 4 4 4 4 4 4 4 4 4 4
## [1] 4 4 4 4 4 4 4 4 4 4
## [1] 4 4 4 4 4 4 4 4 4 4
## [1] 4 4 4 4 4 4 4 4 4 4
## [1] 4 4 4 4 4 4 4 4 4 4
## [1] 4 4 4 4 4 4 4 4 4 4
## [1] 4 4 4 4 4 4 4 4 4 4
## [1] 4 4 4 4 4 4 4 4 4 4
## [1] 4 4 4 4 4 4 4 4 4 4
## [1] 4 4 4 4 4 4 4 4 4 4
## [1] 4 4 4 4 4 4 4 4 4 4
## [1] 4 4 4 4 4 4 4 4 4 4
## [1] 4 4 4 4 4 4 4 4 4 4
## [1] 4 4 4 4 4 4 4 4 4 4
## [1] 4 4 4 4 4 4 4 4 4 4
## [1] 4 4 4 4 4 4 4 4 4 4
## [1] 4 4 4 4 4 4 4 4 4 4
## [1] 4 4 4 4 4 4 4 4 4 4
## [1] 4 4 4 4 4 4 4 4 4 4
## [1] 4 4 4 4 4 4 4 4 4 4
## [1] 4 4 4 4 4 4 4 4 4 4
## [1] 4 4 4 4 4 4 4 4 4 4
## [1] 4 4 4 4 4 4 4 4 4 4
## [1] 4 4 4 4 4 4 4 4 4 4
## [1] 4 4 4 4 4 4 4 4 4 4
## [1] 4 4 4 4 4 4 4 4 4 4
## [1] 4 4 4 4 4 4 4 4 4 4
## [1] 4 4 4 4 4 4 4 4 4 4
## [1] 4 4 4 4 4 4 4 4 4 4
## [1] 4 4 4 4 4 4 4 4 4 4
## [1] 4 4 4 4 4 4 4 4 4 4
## [1] 4 4 4 4 4 4 4 4 4 4
## [1] 4 4 4 4 4 4 4 4 4 4
## [1] 4 4 4 4 4 4 4 4 4 4
## [1] 4 4 4 4 4 4 4 4 4 4
## [1] 4 4 4 4 4 4 4 4 4 4
## [1] 4 4 4 4 4 4 4 4 4 4
## [1] 4 4 4 4 4 4 4 4 4 4
## [1] 4 4 4 4 4 4 4 4 4 4
## [1] 4 4 4 4 4 4 4 4 4 4
## [1] 4 4 4 4 4 4 4 4 4 4
## [1] 4 4 4 4 4 4 4 4 4 4
## [1] 4 4 4 4 4 4 4 4 4 4
## [1] 4 4 4 4 4 4 4 4 4 4
## [1] 4 4 4 4 4 4 4 4 4 4
## [1] 4 4 4 4 4 4 4 4 4 4
## [1] 4 4 4 4 4 4 4 4 4 4
## [1] 4 4 4 4 4 4 4 4 4 4
## [1] 4 4 4 4 4 4 4 4 4 4
## [1] 4 4 4 4 4 4 4 4 4 4
## [1] 4 4 4 4 4 4 4 4 4 4
## [1] 4 4 4 4 4 4 4 4 4 4
## [1] 4 4 4 4 4 4 4 4 4 4
## [1] 4 4 4 4 4 4 4 4 4 4
## [1] 4 4 4 4 4 4 4 4 4 4
## [1] 4 4 4 4 4 4 4 4 4 4

knnsignifs=sum(outpsk<=0.05)/length(outpsk)

knnsignifs

## [1] 0.2362869

### Local Moran's I at Analysis Zone Scale

Local spatial autocorrelation assesses each spatial location for its degree of similarity with adjacent locations. The most common measure is based on Moran's I and is called Local Moran's I and abbreviated as $I_{i}$. Local Moran's I is easily computed for a given location when the values that are to be tested *have already* been z-standardized. However, 'spdep' has no function to undertake significance testing of $I_{i}$ via simulation. As such, the following function was created to undertake Monte Carlo simulation for Local Moran's I at the scale of the 10 analysis zones,

localmoran.mc <- function (invector, inadjmatrix, mlvar=TRUE,nsim = 99, conditional = FALSE){
 require(spdep)

 # z-score the data. See localmoran() mlvar for
 # explanation of this choice of variance
 if (!mlvar)
 z = scale(invector)[, 1]
 else
 z = (invector - mean(invector)) / (sd(invector) * sqrt((length(invector) - 1) / length(invector)))

 # compute the observed local moran's I
 obs = mapply(
 function(X, Y, V, v) {
 V * sum(v[X] * Y)
 },
 X = inadjmatrix$neighbours,
 Y = inadjmatrix$weights,
 V = as.list(z),
 MoreArgs = list(v = z)
 )

 # Create a list of ids for each row so that the current observation can be held constant
 idx = as.list(1:length(invector))

 # repeat the calculation of local I nsim times,
 # for each row calculate Li by shuffling all n-1 data values
 if(conditional){
 refd = replicate(
 nsim,
 mapply(
 function(X, Y, V, v, rid) {
 v[-rid] = sample(v[-rid])
 #gx[,rid]<<-v
 V * sum(v[X] * Y)
 },
 X = inadjmatrix$neighbours,
 Y = inadjmatrix$weights,
 V = as.list(z),
 rid = idx,
 MoreArgs = list(v = z)
 )
 )
 }
 else{
 refd = replicate(
 nsim,
 mapply(
 function(X, Y, V, v, rid) {
 v = sample(v)
 #gx[,rid]<<-v
 V * sum(v[X] * Y)
 },
 X = inadjmatrix$neighbours,
 Y = inadjmatrix$weights,
 V = as.list(z),
 rid = idx,
 MoreArgs = list(v = z)
 )
 )
 }
 res = cbind(obs, refd)
 less = rowSums(sweep(res, 1, res[, 1], "<=")) / (nsim + 1)
 greater = rowSums(sweep(res, 1, res[, 1], ">=")) / (nsim + 1)
 ponesided = apply(cbind(less, greater), 1, min)
 resscale=t(scale(t(res)))
 both = rowSums(
 sweep(abs(resscale),
 1,
 abs(resscale)[, 1],
 ">=")
 ) / (nsim + 1)

 fdrponesided = p.adjustSP(
 ponesided,
 inadjmatrix$neighbours,
 method = "fdr")

 fdrboth = p.adjustSP(
 both,
 inadjmatrix$neighbours,
 method = "fdr")

 cDV = z
 c_mI = as.vector(lag.listw(inadjmatrix, z))

 cDVmean = mean(cDV)
 miMean = mean(c_mI)

 quadrant = vector(mode = "character", length = length(ponesided))
 quadrant[cDV > cDVmean & c_mI > miMean] = "HH"
 quadrant[cDV < cDVmean & c_mI > miMean] = "LH"
 quadrant[cDV > cDVmean & c_mI < miMean] = "HL"
 quadrant[cDV < cDVmean & c_mI < miMean] = "LL"

 # set a statistical significance level for the local Moran's
 signif = 0.05

 # places non-significant Moran's in the category "5"
 quadrant[ponesided > signif] = "NOTSIG"

 quadrant=factor(quadrant,levels=c("HH", "LL", "LH", "HL", "NOTSIG"),ordered=T)


 # Create list of results
 return(list(
 results = data.frame(
 Ii = res[, 1],
 one.sided = ponesided,
 FDRone.sided = fdrponesided,
 two.sided = both,
 FDRtwo.sided = fdrboth,
 quadrant = quadrant
 ),
 raw = res,
 moran.scatter = list(x=cDV,y=c_mI)
 ))
}

Note: Local Moran's I was tested against results from the pysal python [6]. function pysal.esda.moran.Moran_Local(). Interestingly, the means by which python vs. R handle double precision numbers can make a difference in calculating some p-values particularly after 10 decimal places.

Calculate Local Moran's I using conditional randomization for each location,

set.seed(436)

fcpolys.Ii = localmoran.mc(fcpolys.sp$PREV,fcpolys.wt,mlvar=F,nsim = 9999,conditional=T)

fcpolys.Ii$results

## Ii one.sided FDRone.sided two.sided FDRtwo.sided quadrant
## 1 0.324111100 0.1396 0.6980 0.2883 1 NOTSIG
## 2 -0.326109653 0.2162 0.6486 0.3854 1 NOTSIG
## 3 -0.106068222 0.4276 1.0000 0.8542 1 NOTSIG
## 4 0.375099776 0.2436 0.7308 0.4652 1 NOTSIG
## 5 0.266668383 0.1732 0.8660 0.3528 1 NOTSIG
## 6 0.002412039 0.4185 1.0000 0.9147 1 NOTSIG
## 7 0.070270103 0.2013 1.0000 0.3786 1 NOTSIG
## 8 -0.196515464 0.5556 1.0000 1.0000 1 NOTSIG
## 9 0.468076608 0.1303 0.5212 0.2501 1 NOTSIG
## 10 0.241807530 0.4455 0.8910 1.0000 1 NOTSIG

Map significant clusters,

fcpolys.sp$quadrant = fcpolys.Ii$results$quadrant
lcols = c("red", "blue", "skyblue", "coral", "grey90")

spplot(
 fcpolys.sp,
 zcol = "quadrant",
 col.regions = lcols,
 col = "white",
 lwd = 1,
 par.settings = list(axis.line = list(col = 'transparent'))
)

Repeat the above test using the knn=4 nearest neighbor definition of adjacency. Calculate Local Moran's I using conditional randomization for each location,

set.seed(1234)

fcpolysknn.Ii = localmoran.mc(fcpolys.sp$PREV,fcpolys.kwt,mlvar=F,nsim = 999,conditional=T)

fcpolysknn.Ii$results

## Ii one.sided FDRone.sided two.sided FDRtwo.sided quadrant
## 1 0.10092191 0.247 1.000 0.496 1.000 NOTSIG
## 2 -0.24227818 0.124 0.620 0.246 1.000 NOTSIG
## 3 -0.25735107 0.230 1.000 0.423 1.000 NOTSIG
## 4 0.58208635 0.014 0.070 0.031 0.155 LL
## 5 -0.12475446 0.448 1.000 0.872 1.000 NOTSIG
## 6 -0.04491266 0.285 1.000 0.547 1.000 NOTSIG
## 7 0.07027010 0.181 0.905 0.346 1.000 NOTSIG
## 8 0.07949290 0.267 1.000 0.545 1.000 NOTSIG
## 9 -0.26933930 0.359 1.000 0.773 1.000 NOTSIG
## 10 -1.07104678 0.041 0.205 0.091 0.455 HL

## Postcode District scale analysis

Load Data and libraries

localmoranfilepath = "C:/Users/laggi/OneDrive/Lisa_GIS/Revision/DATA/shapefiles/"
rid = "OBJECTID"
smallerScaleVar = "PREV"
largerScaleVar = "PREV_1"

# Shapefile SRS
coordsys = CRS("+init=epsg:32630")

bivar.shp = paste(localmoranfilepath, "bivar2.shp", sep = "")

# 312 postcode districts with PP and PP at 10 analysis zone scale
fcpolysbi.sp = readShapePoly(bivar.shp, proj4string = coordsys, repair =
T)

Create queen's case neighbor object,

fcpolysbi.nb = poly2nb(fcpolysbi.sp, row.names = fcpolysbi.sp$data[[rid]], queen =
 TRUE)

Assess neighbor summary,

summary(fcpolysbi.nb)

## Neighbour list object:
## Number of regions: 312
## Number of nonzero links: 1470
## Percentage nonzero weights: 1.510108
## Average number of links: 4.711538
## 4 regions with no links:
## 85 127 253 302
## Link number distribution:
##
## 0 1 2 3 4 5 6 7 8 9 10 11 12
## 4 17 30 47 46 58 46 33 19 5 4 2 1
## 17 least connected regions:
## 115 119 143 155 164 166 171 178 179 185 186 249 269 270 271 303 304 with 1 link
## 1 most connected region:
## 188 with 12 links

Examine neighbor graph to determine missing links,

plot(fcpolysbi.sp,col='whitesmoke',border='grey80')

plot.nb(
 fcpolysbi.nb,
 coordinates(fcpolysbi.sp),
 col = 2,
 pch = 16,
 add = T
)

There are 4 disconnected regions. Using information on ferry routes between the outer Hebrides and Orkney and the mainland, we modified the queen's case adjacency matrix to add these additional links. In some cases, links are between different parts of the mainland as well in the inner Hebrides.

One disconnected region KA27 (postcode district Isle of Arran) is made adjacent to PA29 (Tarbert) (ferry from Lochranza to Tarbert), KA22 (Androssan) (ferry from Lamlash to Androssan) and PA28 (Ferry from Lamlash to Campbeltown).

fcpolysbi.sp$name[c(86,120, 240, 83)]

## [1] KA27 PA28 PA29 KA22
## 312 Levels: DG1 DG10 DG11 DG12 DG13 DG14 DG16 DG2 DG3 DG4 DG5 DG6 ... PH9

fcpolysbi.nb[[86]]=as.integer(c(83, 120, 240))

fcpolysbi.nb[[83]]=as.integer(sort(c(86,fcpolysbi.nb[[83]])))
fcpolysbi.nb[[120]]=as.integer(sort(c(86,fcpolysbi.nb[[120]])))
fcpolysbi.nb[[240]]=as.integer(sort(c(86,fcpolysbi.nb[[240]])))

One disconnected region PA61 (Island Colonsay) is made adjacent to PA46 (Ferry from Skalasaig to Port Askaig) and to PA34 (ferry from Skalasaig to Oban),

fcpolysbi.sp$name[c(254, 242, 249)]

## [1] PA61 PA34 PA46
## 312 Levels: DG1 DG10 DG11 DG12 DG13 DG14 DG16 DG2 DG3 DG4 DG5 DG6 ... PH9

fcpolysbi.nb[[254]]=as.integer(c(242, 249))

fcpolysbi.nb[[242]]=as.integer(sort(c(254,fcpolysbi.nb[[242]])))
fcpolysbi.nb[[249]]=as.integer(sort(c(254,fcpolysbi.nb[[249]])))

Next, the Isle of Gigha PA41 was made adjacent to PA29 (Ferry from Ardminish to Tayinloan),

fcpolysbi.sp$name[c(128, 240)]

## [1] PA41 PA29
## 312 Levels: DG1 DG10 DG11 DG12 DG13 DG14 DG16 DG2 DG3 DG4 DG5 DG6 ... PH9

fcpolysbi.nb[[128]]=as.integer(c(240))

fcpolysbi.nb[[240]]=as.integer(sort(c(128,fcpolysbi.nb[[240]])))

Finally, the Isle of Egg, PH42 was made adjacent to PH42 (ferry from Galamsdale to Kinloch) and PH41 (ferry from Isle of Muck to Glamisdale and to Mallaig)

fcpolysbi.sp$name[c(303, 302, 304)]

## [1] PH42 PH41 PH43
## 312 Levels: DG1 DG10 DG11 DG12 DG13 DG14 DG16 DG2 DG3 DG4 DG5 DG6 ... PH9

fcpolysbi.nb[[303]]=as.integer(c(302,304))

fcpolysbi.nb[[302]]=as.integer(sort(c(303,fcpolysbi.nb[[302]])))
fcpolysbi.nb[[304]]=as.integer(sort(c(303,fcpolysbi.nb[[304]])))
fcpolysbi.nb[[304]]=as.integer(sort(c(302,fcpolysbi.nb[[304]])))

In addition, Island district postcodes were connected to mainland district postcodes using ferry routes,

HS1 was connected to IV26 (ferry from Stornaway to Ullapool)

fcpolysbi.sp$name[c(165, 67)]

## [1] HS1 IV26
## 312 Levels: DG1 DG10 DG11 DG12 DG13 DG14 DG16 DG2 DG3 DG4 DG5 DG6 ... PH9

fcpolysbi.nb[[165]]=as.integer(sort(c(67,fcpolysbi.nb[[165]])))
fcpolysbi.nb[[67]]=as.integer(sort(c(165,fcpolysbi.nb[[67]])))

HS3 was connected to IV51 (ferry from Tarbert to Uig)

fcpolysbi.sp$name[c(166, 200)]

## [1] HS3 IV51
## 312 Levels: DG1 DG10 DG11 DG12 DG13 DG14 DG16 DG2 DG3 DG4 DG5 DG6 ... PH9

fcpolysbi.nb[[166]]=as.integer(sort(c(200,fcpolysbi.nb[[166]])))
fcpolysbi.nb[[200]]=as.integer(sort(c(166,fcpolysbi.nb[[200]])))

HS6 was connected to IV51 (ferry from Lochmaddy to Uig)

fcpolysbi.sp$name[c(169, 200)]

## [1] HS6 IV51
## 312 Levels: DG1 DG10 DG11 DG12 DG13 DG14 DG16 DG2 DG3 DG4 DG5 DG6 ... PH9

fcpolysbi.nb[[169]]=as.integer(sort(c(200,fcpolysbi.nb[[169]])))
fcpolysbi.nb[[200]]=as.integer(sort(c(169,fcpolysbi.nb[[200]])))

HS8 was connected to PH41 (ferry from Lochboisdale to Mallaig),

fcpolysbi.sp$name[c(171, 302)]

## [1] HS8 PH41
## 312 Levels: DG1 DG10 DG11 DG12 DG13 DG14 DG16 DG2 DG3 DG4 DG5 DG6 ... PH9

fcpolysbi.nb[[171]]=as.integer(sort(c(302,fcpolysbi.nb[[171]])))
fcpolysbi.nb[[302]]=as.integer(sort(c(171,fcpolysbi.nb[[302]])))

HS9 was connected to PA34 (ferry from Castlebay to Oban),

fcpolysbi.sp$name[c(172, 242)]

## [1] HS9 PA34
## 312 Levels: DG1 DG10 DG11 DG12 DG13 DG14 DG16 DG2 DG3 DG4 DG5 DG6 ... PH9

fcpolysbi.nb[[172]]=as.integer(sort(c(242,fcpolysbi.nb[[172]])))
fcpolysbi.nb[[242]]=as.integer(sort(c(172,fcpolysbi.nb[[242]])))

IV45 was connected to PH41 (ferry from Armadale to Mallaig),

fcpolysbi.sp$name[c(302, 194)]

## [1] PH41 IV45
## 312 Levels: DG1 DG10 DG11 DG12 DG13 DG14 DG16 DG2 DG3 DG4 DG5 DG6 ... PH9

fcpolysbi.nb[[302]]=as.integer(sort(c(194,fcpolysbi.nb[[302]])))
fcpolysbi.nb[[194]]=as.integer(sort(c(302,fcpolysbi.nb[[194]])))

IV48 was connected to IV40 (ferry from Sconser to Inverarish),

fcpolysbi.sp$name[c(197, 189)]

## [1] IV48 IV40
## 312 Levels: DG1 DG10 DG11 DG12 DG13 DG14 DG16 DG2 DG3 DG4 DG5 DG6 ... PH9

fcpolysbi.nb[[197]]=as.integer(sort(c(189,fcpolysbi.nb[[197]])))
fcpolysbi.nb[[189]]=as.integer(sort(c(197,fcpolysbi.nb[[189]])))

PA65 was connected to PA80 (ferry from Fishnish to Lochaline),

fcpolysbi.sp$name[c(258, 273)]

## [1] PA65 PA80
## 312 Levels: DG1 DG10 DG11 DG12 DG13 DG14 DG16 DG2 DG3 DG4 DG5 DG6 ... PH9

fcpolysbi.nb[[258]]=as.integer(sort(c(273,fcpolysbi.nb[[258]])))
fcpolysbi.nb[[273]]=as.integer(sort(c(258,fcpolysbi.nb[[273]])))

PA75 was connected to PH36 (ferry from Tobermory to Killchoan),

fcpolysbi.sp$name[c(269, 296)]

## [1] PA75 PH36
## 312 Levels: DG1 DG10 DG11 DG12 DG13 DG14 DG16 DG2 DG3 DG4 DG5 DG6 ... PH9

fcpolysbi.nb[[269]]=as.integer(sort(c(296,fcpolysbi.nb[[269]])))
fcpolysbi.nb[[296]]=as.integer(sort(c(269,fcpolysbi.nb[[296]])))

PA42 was connected to PA29 (ferry from Port Ellen to Kennacraig),

fcpolysbi.sp$name[c(245, 240)]

## [1] PA42 PA29
## 312 Levels: DG1 DG10 DG11 DG12 DG13 DG14 DG16 DG2 DG3 DG4 DG5 DG6 ... PH9

fcpolysbi.nb[[245]]=as.integer(sort(c(240,fcpolysbi.nb[[245]])))
fcpolysbi.nb[[240]]=as.integer(sort(c(245,fcpolysbi.nb[[240]])))

PA42 was connected to PA29 (ferry from Port Askaig to Kennacraig),

fcpolysbi.sp$name[c(249, 240)]

## [1] PA46 PA29
## 312 Levels: DG1 DG10 DG11 DG12 DG13 DG14 DG16 DG2 DG3 DG4 DG5 DG6 ... PH9

fcpolysbi.nb[[249]]=as.integer(sort(c(240,fcpolysbi.nb[[249]])))
fcpolysbi.nb[[240]]=as.integer(sort(c(249,fcpolysbi.nb[[240]])))

PA21 was connected to PA29 (ferry from Portavadie to Tarbert),

fcpolysbi.sp$name[c(234, 240)]

## [1] PA21 PA29
## 312 Levels: DG1 DG10 DG11 DG12 DG13 DG14 DG16 DG2 DG3 DG4 DG5 DG6 ... PH9

fcpolysbi.nb[[234]]=as.integer(sort(c(240,fcpolysbi.nb[[234]])))
fcpolysbi.nb[[240]]=as.integer(sort(c(234,fcpolysbi.nb[[240]])))

PA20 was connected to PA17 (ferry from Rothesay to Skelmorlie),

fcpolysbi.sp$name[c(232, 118)]

## [1] PA17 PA20
## 312 Levels: DG1 DG10 DG11 DG12 DG13 DG14 DG16 DG2 DG3 DG4 DG5 DG6 ... PH9

fcpolysbi.nb[[232]]=as.integer(sort(c(118,fcpolysbi.nb[[232]])))
fcpolysbi.nb[[118]]=as.integer(sort(c(232,fcpolysbi.nb[[118]])))

PA78 was connected to PA34 (ferry from Coll to Oban),

fcpolysbi.sp$name[c(242, 272)]

## [1] PA34 PA78
## 312 Levels: DG1 DG10 DG11 DG12 DG13 DG14 DG16 DG2 DG3 DG4 DG5 DG6 ... PH9

fcpolysbi.nb[[242]]=as.integer(sort(c(272,fcpolysbi.nb[[242]])))
fcpolysbi.nb[[272]]=as.integer(sort(c(242,fcpolysbi.nb[[272]])))

PA77 was connected to HS9 (ferry from Castlebay to Tiree),

fcpolysbi.sp$name[c(271, 172)]

## [1] PA77 HS9
## 312 Levels: DG1 DG10 DG11 DG12 DG13 DG14 DG16 DG2 DG3 DG4 DG5 DG6 ... PH9

fcpolysbi.nb[[271]]=as.integer(sort(c(172,fcpolysbi.nb[[271]])))
fcpolysbi.nb[[172]]=as.integer(sort(c(271,fcpolysbi.nb[[172]])))

In the orkney's KW17 was connected to KW1 (ferry from St. Margarets Hope to Cainsbay),

fcpolysbi.sp$name[c(95, 222)]

## [1] KW1 KW17
## 312 Levels: DG1 DG10 DG11 DG12 DG13 DG14 DG16 DG2 DG3 DG4 DG5 DG6 ... PH9

fcpolysbi.nb[[95]]=as.integer(sort(c(222,fcpolysbi.nb[[95]])))
fcpolysbi.nb[[222]]=as.integer(sort(c(95,fcpolysbi.nb[[222]])))

KW16 was connected to KW14 (ferry from Scrabster to Stromness),

fcpolysbi.sp$name[c(221, 97)]

## [1] KW16 KW14
## 312 Levels: DG1 DG10 DG11 DG12 DG13 DG14 DG16 DG2 DG3 DG4 DG5 DG6 ... PH9

fcpolysbi.nb[[97]]=as.integer(sort(c(221,fcpolysbi.nb[[97]])))
fcpolysbi.nb[[221]]=as.integer(sort(c(97,fcpolysbi.nb[[221]])))

Re-examine the neighbor graph,

plot(fcpolysbi.sp,col='whitesmoke',border='grey80')


plot.nb(
 fcpolysbi.nb,
 coordinates(fcpolysbi.sp),
 col = 2,
 pch = 16,
 add = T
)

Show summary of neighbor object,

summary(fcpolysbi.nb)

## Neighbour list object:
## Number of regions: 312
## Number of nonzero links: 1521
## Percentage nonzero weights: 1.5625
## Average number of links: 4.875
## Link number distribution:
##
## 1 2 3 4 5 6 7 8 9 10 11 13
## 12 29 50 48 59 47 34 18 6 5 3 1
## 12 least connected regions:
## 115 127 143 155 166 178 179 185 186 249 269 304 with 1 link
## 1 most connected region:
## 188 with 13 links

Convert to row-standardized weights matrix,

fcpolysbi.wt = nb2listw(fcpolysbi.nb, style = "W", zero.policy = TRUE)

### Global Moran's I Analysis

First, test global Moran's I to determine if there is significant global autocorrelation at the postcode district level,

set.seed(988)

moran.mc(fcpolysbi.sp$PREV,fcpolysbi.wt,999)

##
## Monte-Carlo simulation of Moran I
##
## data: fcpolysbi.sp$PREV
## weights: fcpolysbi.wt
## number of simulations + 1: 1000
##
## statistic = 0.041194, observed rank = 922, p-value = 0.078
## alternative hypothesis: greater

To assess the sensitivity of this result to the number of neighbors, we undertake the same test using a constant adjacency matrix where each analysis zone is connected to its four nearest neighbors (k=4) using euclidean distance,

fcpolysbi.kn = knearneigh(coordinates(fcpolysbi.sp),k=4)

Next, the function knn2nb() creates the neighbor matrix (knn=4),

fcpolysbi.knb = knn2nb(fcpolysbi.kn,row.names = fcpolysbi.sp$name)

plot(fcpolysbi.sp,col='whitesmoke',border='grey80')

plot.nb(fcpolysbi.knb,coordinates(fcpolysbi.sp),col=2,pch=16,add=T)

Print summary of neighbor object,

summary(fcpolysbi.knb)

## Neighbour list object:
## Number of regions: 312
## Number of nonzero links: 1248
## Percentage nonzero weights: 1.282051
## Average number of links: 4
## Non-symmetric neighbours list
## Link number distribution:
##
## 4
## 312
## 312 least connected regions:
## DG11 DG2 DG4 DG5 DG7 FK1 FK10 FK14 FK2 FK21 FK7 FK8 FK9 G11 G12 G13 G14 G15 G20 G22 G23 G3 G31 G32 G33 G4 G40 G41 G42 G43 G44 G45 G46 G5 G51 G52 G53 G61 G62 G63 G64 G65 G66 G67 G68 G69 G71 G72 G73 G74 G75 G76 G77 G78 G81 G82 G83 G84 HS2 IV10 IV12 IV13 IV18 IV19 IV2 IV24 IV26 IV27 IV3 IV36 IV6 KA1 KA10 KA11 KA12 KA13 KA15 KA17 KA18 KA19 KA2 KA21 KA22 KA23 KA24 KA27 KA3 KA30 KA4 KA5 KA6 KA7 KA8 KA9 KW1 KW10 KW14 KW9 ML1 ML10 ML11 ML12 ML2 ML3 ML5 ML6 ML7 ML9 PA1 PA11 PA12 PA13 PA14 PA15 PA16 PA19 PA2 PA20 PA23 PA28 PA3 PA30 PA31 PA32 PA35 PA37 PA4 PA41 PA5 PA6 PA8 PA9 PH16 PH22 PH25 PH3 DG1 DG10 DG12 DG13 DG14 DG16 DG3 DG6 DG8 DG9 FK11 FK12 FK13 FK15 FK16 FK17 FK18 FK19 FK20 FK3 FK4 FK5 FK6 G1 G2 G21 G34 G60 HS1 HS3 HS4 HS5 HS6 HS7 HS8 HS9 IV1 IV11 IV14 IV15 IV16 IV17 IV20 IV21 IV22 IV23 IV25 IV28 IV30 IV31 IV32 IV4 IV40 IV41 IV42 IV43 IV44 IV45 IV46 IV47 IV48 IV49 IV5 IV51 IV52 IV53 IV54 IV55 IV56 IV63 IV7 IV8 IV9 KA14 KA16 KA20 KA25 KA26 KA28 KA29 KW11 KW12 KW13 KW15 KW16 KW17 KW2 KW3 KW5 KW6 KW7 KW8 ML4 ML8 PA10 PA17 PA18 PA21 PA22 PA24 PA25 PA26 PA27 PA29 PA33 PA34 PA36 PA38 PA42 PA43 PA44 PA45 PA46 PA47 PA48 PA49 PA60 PA61 PA62 PA63 PA64 PA65 PA66 PA67 PA68 PA69 PA7 PA70 PA71 PA72 PA73 PA74 PA75 PA76 PA77 PA78 PA80 PH1 PH10 PH11 PH12 PH13 PH14 PH15 PH17 PH18 PH19 PH2 PH20 PH21 PH23 PH24 PH26 PH30 PH31 PH32 PH33 PH34 PH35 PH36 PH37 PH38 PH39 PH4 PH40 PH41 PH42 PH43 PH44 PH49 PH5 PH50 PH6 PH7 PH8 PH9 with 4 links
## 312 most connected regions:
## DG11 DG2 DG4 DG5 DG7 FK1 FK10 FK14 FK2 FK21 FK7 FK8 FK9 G11 G12 G13 G14 G15 G20 G22 G23 G3 G31 G32 G33 G4 G40 G41 G42 G43 G44 G45 G46 G5 G51 G52 G53 G61 G62 G63 G64 G65 G66 G67 G68 G69 G71 G72 G73 G74 G75 G76 G77 G78 G81 G82 G83 G84 HS2 IV10 IV12 IV13 IV18 IV19 IV2 IV24 IV26 IV27 IV3 IV36 IV6 KA1 KA10 KA11 KA12 KA13 KA15 KA17 KA18 KA19 KA2 KA21 KA22 KA23 KA24 KA27 KA3 KA30 KA4 KA5 KA6 KA7 KA8 KA9 KW1 KW10 KW14 KW9 ML1 ML10 ML11 ML12 ML2 ML3 ML5 ML6 ML7 ML9 PA1 PA11 PA12 PA13 PA14 PA15 PA16 PA19 PA2 PA20 PA23 PA28 PA3 PA30 PA31 PA32 PA35 PA37 PA4 PA41 PA5 PA6 PA8 PA9 PH16 PH22 PH25 PH3 DG1 DG10 DG12 DG13 DG14 DG16 DG3 DG6 DG8 DG9 FK11 FK12 FK13 FK15 FK16 FK17 FK18 FK19 FK20 FK3 FK4 FK5 FK6 G1 G2 G21 G34 G60 HS1 HS3 HS4 HS5 HS6 HS7 HS8 HS9 IV1 IV11 IV14 IV15 IV16 IV17 IV20 IV21 IV22 IV23 IV25 IV28 IV30 IV31 IV32 IV4 IV40 IV41 IV42 IV43 IV44 IV45 IV46 IV47 IV48 IV49 IV5 IV51 IV52 IV53 IV54 IV55 IV56 IV63 IV7 IV8 IV9 KA14 KA16 KA20 KA25 KA26 KA28 KA29 KW11 KW12 KW13 KW15 KW16 KW17 KW2 KW3 KW5 KW6 KW7 KW8 ML4 ML8 PA10 PA17 PA18 PA21 PA22 PA24 PA25 PA26 PA27 PA29 PA33 PA34 PA36 PA38 PA42 PA43 PA44 PA45 PA46 PA47 PA48 PA49 PA60 PA61 PA62 PA63 PA64 PA65 PA66 PA67 PA68 PA69 PA7 PA70 PA71 PA72 PA73 PA74 PA75 PA76 PA77 PA78 PA80 PH1 PH10 PH11 PH12 PH13 PH14 PH15 PH17 PH18 PH19 PH2 PH20 PH21 PH23 PH24 PH26 PH30 PH31 PH32 PH33 PH34 PH35 PH36 PH37 PH38 PH39 PH4 PH40 PH41 PH42 PH43 PH44 PH49 PH5 PH50 PH6 PH7 PH8 PH9 with 4 links

You can see that 312 polygons all have 4 neighbors. Finally, the neighbor object is converted to a spatial weights matrix,

fcpolysbi.kwt = nb2listw(fcpolysbi.knb, style = "W", zero.policy = FALSE)

Undertake global Moran's I test as in the previous for the Queen's + ferry routes,

set.seed(542)

fcpolysbi.ktest = moran.mc(
 fcpolysbi.sp$PREV,
 fcpolysbi.kwt,
 nsim = 999,
 alternative="greater")

fcpolysbi.ktest

##
## Monte-Carlo simulation of Moran I
##
## data: fcpolysbi.sp$PREV
## weights: fcpolysbi.kwt
## number of simulations + 1: 1000
##
## statistic = 0.054047, observed rank = 949, p-value = 0.051
## alternative hypothesis: greater

The Moran's I test yields a $p$-value = 0.051. As such, for the Monte-Carlo simulation of Moran I, the null hypothesis of no spatial auto-correlation for VS prevalence within the 10 analysis zones is accepted.

### Local Moran's I for PP within the postcode districts

First, compute the local measure using our definition of spatial dependency based on Ferry routes and Queen's case adjacency,

set.seed(65)
fcpolysbi.Ii = localmoran.mc(fcpolysbi.sp$PREV,fcpolysbi.wt,mlvar=F,nsim = 9999,conditional = T)

head(fcpolysbi.Ii$results)

## Ii one.sided FDRone.sided two.sided FDRtwo.sided quadrant
## 1 -0.060472363 0.0153 0.1224 0.1130 0.904 HL
## 2 -0.005466036 0.3497 1.0000 0.5031 1.000 NOTSIG
## 3 -0.001989205 0.3334 1.0000 0.9659 1.000 NOTSIG
## 4 -0.006066452 0.3729 1.0000 0.8743 1.000 NOTSIG
## 5 0.013999994 0.4846 1.0000 0.7399 1.000 NOTSIG
## 6 0.052960568 0.3243 1.0000 0.4750 1.000 NOTSIG

Create a Moran scatterplot to examine locations of regions with high leverage,

# Place the scatter plot variables in two vectors

x = fcpolysbi.Ii$moran.scatter$x
y = fcpolysbi.Ii$moran.scatter$y

# Get the text quadrant values in a vector
quads = fcpolysbi.Ii$results$quadrant

# Make a blank plot of x,y
plot(x,y, type ="n",xlim=c(-2,15),ylim=c(-2,3),ylab = "Spatially lagged prevalence",xlab = "Prevalence")

# Add the average x,y values as lines
abline(v = 0)
abline(h = 0)

# Create a vector of row labels equal to the number of polygons or regions
labs = fcpolysbi.sp$BUID

# Plot the row labels as text in red if significant and black if not significant
text(x[quads == "NOTSIG"],
 y[quads == "NOTSIG"],
 labs,
 cex = 0.5)

text(x[quads != "NOTSIG"],
 y[quads != "NOTSIG"],
 labs[quads != "NOTSIG"],
 cex = 0.75,
 col = 2)

# Label the quadrants by first resetting the plot space to the unit square for convienence of placing labels
par(usr = c(0, 1, 0, 1))
text(
 c(0.1, 0.9, 0.9, 0.1),
 c(.9, .9, .1, .1),
 c("LH", "HH", "HL", "LL"),
 col = 4,
 cex = 2
)

Plot the reference distributions for significant $I_{i}$ values,

sub=(1:length(fcpolysbi.Ii$results$Ii))[fcpolysbi.Ii$results$one.sided<=0.05]
nplots=ceiling(sqrt(length(sub)))
par(mfrow=c(nplots,nplots))

for (i in 1:length(sub)){
 rw = sub[i]
 hist(fcpolysbi.Ii$raw[rw,],main=rw)
 abline(v=fcpolysbi.Ii$raw[rw,1])

}

Plot the results,

fcpolysbi.sp$quadrantii = fcpolysbi.Ii$results$quadrant
lcols = c("red", "blue", "skyblue", "coral", "grey90")

spplot(
 fcpolysbi.sp,
 zcol = "quadrantii",
 col.regions = lcols,
 col = "white",
 lwd = 1,
 par.settings = list(axis.line = list(col = 'transparent'))
)

Write to file for mapping in ArcGIS,

library(maptools)
outpath = 'C:/Users/laggi/OneDrive/Lisa_GIS/Revision/DATA/URICTa'
fcpolysbi.sp@data$quadrant=factor(unclass(fcpolysbi.sp@data$quadrant))
fcpolysbi.sp@data$quadrantii=factor(unclass(fcpolysbi.sp@data$quadrantii))
writeSpatialShape(fcpolysbi.sp,outpath)

### Bivariate Local spatial autocorrelation for PP from district-zone

Traditionally, bivariate local Moran's I has been used to explore relations between a spatial unit's value at one time and its neighboring values at a second time period. Here we use the method of Nelson et Brewer (2017) to test the stability of the relation between prevalence at the postcode district scale and the coarser scale of the analysis zones [7]. In this case, our interest is in understanding the relation between period prevalence at the postcode district scale and period prevalence at the aggregate zone scale in order to address the question: Is PP at a given postcode district significantly different or similar to the zone level prevalence in surrounding units? In this analysis we use bi-variate Local Moran's I in the manner described by Nelson and Brewer (2017) to explore stability of PP across the spatial scales in the exploratory study [7]. While high-low Significant finding indicates that the post code district in question is a hotspot at the zone level etc.

The following two R functions were written to undertake bivariate local Moran's I tests using a Monte-Carlo randomization approach that is conditional on PP remaining constant at the postcode district scale:

localmoran.bivar = function(s0=fcpolysbi.sp$PREV, s1=fcpolysbi.sp$PREV_1, W=fcpolysbi.wt, signif=0.05,nsim=999){
 # Default function arguments are for this particular dataset with PP at the postcode district scale (PREV) and
 # PP at the analysis zone scale (PREV_1 in the dataset)

 # Store observed bivariate local moran's I values
 lmvals=localmoranb(s0,s1,W,mlvar=FALSE)

 # Add observed as one simulation
 nsim=nsim+1

 sim.I=matrix(0,nsim,length(s0))

 # Include observed in simulation as first row
 sim.I[1,]=lmvals

 # Undertake simulation using localmoranb.mc function and permuting
 # s1 at each iteration (mirrors the way GeoDa does this) by keeping
 # s0 constant at each iteration

 for(i in 2:nsim){

 sim.I[i,]=localmoranb(s0,sample(s1),W,mlvar=FALSE)

 }

 # Calculate 1-sided p-values for less and greater (pos/neg SA)
 less = colSums(sweep(sim.I, 2, sim.I[1, ], "<=")) / (nsim)
 greater = colSums(sweep(sim.I, 2, sim.I[1, ], ">=")) / (nsim)
 ponesided = apply(cbind(less, greater), 1, min)

 # Normalize
 z = scale(s0)[, 1] #e.g,. time 0 bivar local I
 z1 = scale(s1)[, 1] # time 1

 # Create bivariate moran scatterplot to get quadrants for High-High,High-Low etc..
 cDV = z
 c_mI = as.vector(lag.listw(W, z1))

 # Ensure NAs are set to zero in lagged list
 #message("NAs in lagged values set to zero.")
 c_mI[is.na(c_mI)]=0

 # bivariate moran's I global same as GeoDa
 lmoranglobalbivar = lm(c_mI~cDV)$coefficients[2]

 cDVmean = mean(cDV)
 miMean = mean(c_mI)

 quadrant = vector(mode = "character", length = length(ponesided))
 quadrant[cDV > cDVmean & c_mI > miMean] = "HH"
 quadrant[cDV < cDVmean & c_mI > miMean] = "LH"
 quadrant[cDV > cDVmean & c_mI < miMean] = "HL"
 quadrant[cDV < cDVmean & c_mI < miMean] = "LL"

 # places non-significant Moran's in the category "5"
 quadrant[ponesided > signif] = "NOTSIG"
 quadrant[ponesided > signif] = "NOTSIG"

 quadrant=factor(quadrant,levels=c("HH", "LL", "LH", "HL", "NOTSIG"),ordered=T)

 x = cDV
 y = c_mI

 return(list(results=data.frame(Ii=lmvals,quadrant=quadrant,less=less,greater=greater,ponesided=ponesided),global=lmoranglobalbivar,raw=sim.I))


}

# Bivariate local moran's I function
localmoranb = function (invector,invector2, inadjmatrix, mlvar=TRUE){
 require(spdep)

 # z-score the data. See localmoran() mlvar for
 # explanation of this choice of variance
 if (!mlvar){
 z = scale(invector)[, 1]#time 0
 z1 = scale(invector2)[, 1]#time 1
 }
 else{
 z = (invector - mean(invector)) / (sd(invector) * sqrt((length(invector) - 1) / length(invector)))
 z1 = (invector2 - mean(invector2)) / (sd(invector2) * sqrt((length(invector2) - 1) / length(invector2)))
 }
 # compute bivariate local moran's I
 obs = mapply(
 function(X, Y, V, v) {
 V * sum(v[X] * Y)
 },
 X = inadjmatrix$neighbours,
 Y = inadjmatrix$weights,
 V = as.list(z),
 MoreArgs = list(v = z1)
 )


 return(obs)
}

Run the default simulation for bivariate local Moran's I using the above data set,

set.seed(843)

fcpolys.resbi=localmoran.bivar(nsim=9999)

Plot the reference distributions for significant Ii values,

subbivar=(1:length(fcpolys.resbi$results$ponesided))[fcpolys.resbi$results$ponesided<=0.05]
nplotsbv=ceiling(sqrt(length(subbivar)))
par(mfrow=c(nplotsbv,nplotsbv))

for (i in 1:length(subbivar)){
 hist(fcpolys.resbi$raw[,subbivar[i]],main=subbivar[i])
 abline(v=fcpolys.resbi$raw[1,subbivar[i]],col=2)

}

Plot the results,

fcpolysbi.sp$quadrant = fcpolys.resbi$results$quadrant
lcols = c("grey30", "grey50", "lightskyblue", "red", "grey90")

spplot(
 fcpolysbi.sp,
 zcol = "quadrant",
 col.regions = lcols,
 col = "white",
 lwd = 1,
 par.settings = list(axis.line = list(col = 'transparent'))
) + layer(sp.polygons(fcpolys.sp, lwd=2))

#par(mfrow=c(1,2))

fcpolysbi.sp$quadrantii = fcpolysbi.Ii$results$quadrant
lcols = c("red", "blue", "skyblue", "coral", "grey90")

one=spplot(
 fcpolysbi.sp,
 zcol = "quadrantii",
 col.regions = lcols,
 col = "grey80",
 lwd = 1,
 par.settings = list(axis.line = list(col = 'transparent')))

library(latticeExtra)
fcpolysbi.sp$quadrant = fcpolys.resbi$results$quadrant
lcols = c("grey30", "grey50", "magenta", "green", "grey90")

two=spplot(
 fcpolysbi.sp,
 zcol = "quadrant",
 col.regions = lcols,
 col = "grey80",
 lwd = 1,
 par.settings = list(axis.line = list(col = 'transparent'))
) + layer(sp.polygons(fcpolys.sp, lwd=2))

print(one, split=c(1,1,2,1),more=T)
print(two, split=c(2,1,2,1))


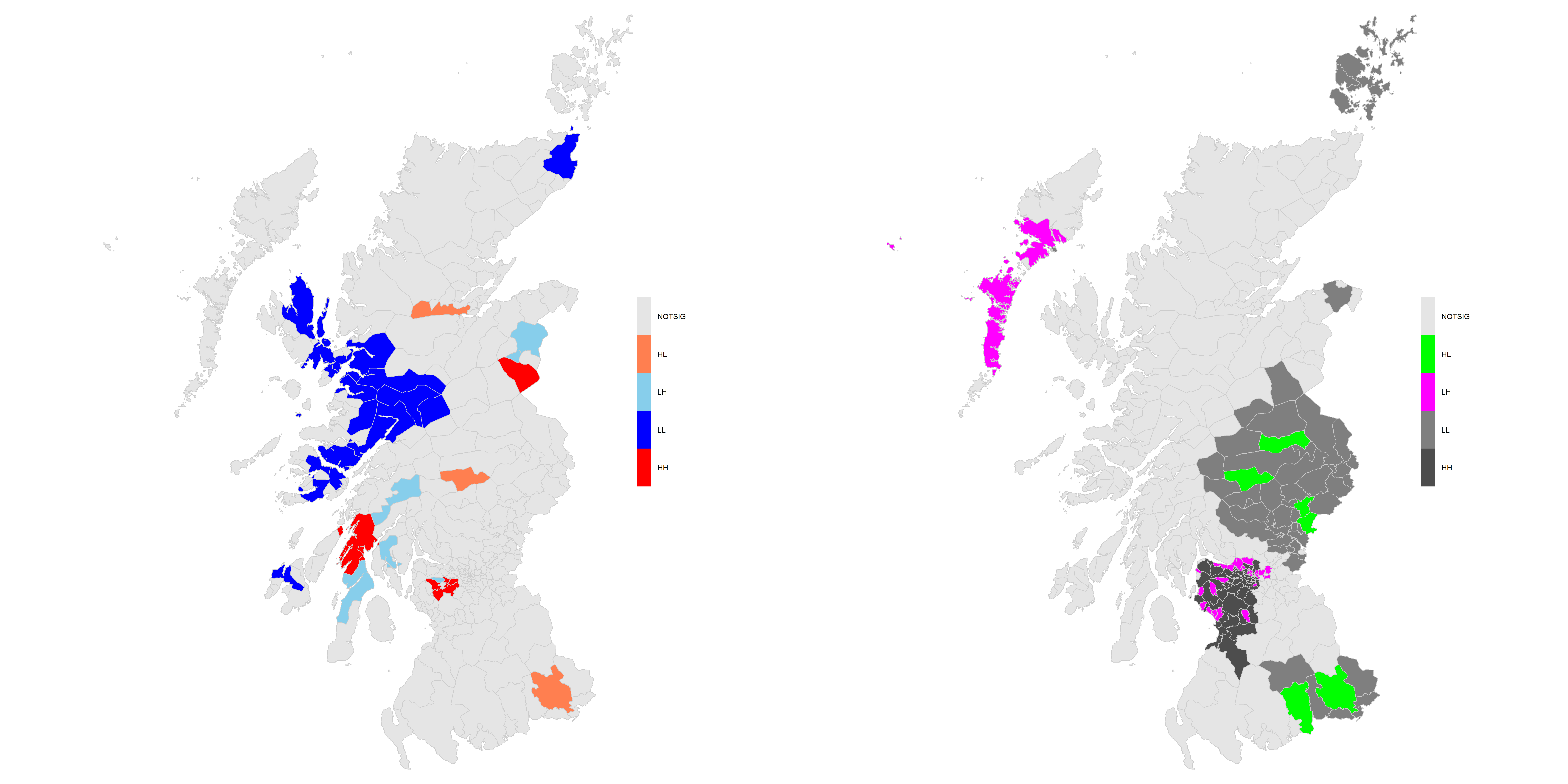


Export as spatial data layer for mapping in ArcGIS

library(maptools)
outpath = 'C:/Users/laggi/OneDrive/Lisa_GIS/Revision/DATA/bivarresults2'

fcpolysbi.sp$Ii=fcpolys.resbi$results$Ii
fcpolysbi.sp$pval=fcpolys.resbi$results$ponesided

fcpolysbi.sp@data$quadrant=factor(unclass(fcpolysbi.sp@data$quadrant))

writeSpatialShape(fcpolysbi.sp,outpath)

Compare bivariate local Moran's I results for spatial dependency based on 4 nearest neighbors to the custom spatial dependency scheme described above.

set.seed(854)
fcpolys.resbik=localmoran.bivar(nsim=9999,W=fcpolysbi.kwt)

Plot the results,

fcpolysbi.sp$quadrantk = fcpolys.resbik$results$quadrant
lcols = c("red", "blue", "skyblue", "coral", "grey90")

spplot(
 fcpolysbi.sp,
 zcol = c("quadrant","quadrantk"),
 col.regions = lcols,
 col = "white",
 lwd = 1,
 par.settings = list(axis.line = list(col = 'transparent'),mar = c(0,0,0,0),mai=c(0,0,0,0),
 pty = "m",
 xaxs = "i",
 xaxt = 'n',
 xpd = FALSE,
 yaxs = "i",
 yaxt = 'n')
)

Overall, the results of the bivariate Moran's I for the the knn=4 nearest neighbor definition of spatial dependency are similar to those based on our defined adjacency matrix using Ferry routes and Queen's case.

**Figures:**

Figure 1X:

library(spdep)
par(mfrow=c(1,2),mai=c(0,0,0,0))

plot(fcpolys.sp,col='whitesmoke',border='grey80')

plot.nb(fcpolys.nb,coordinates(fcpolys.sp),col=2,pch=16,add=T)

text(coordinates(fcpolys.sp)[,1],coordinates(fcpolys.sp)[,2],fcpolys.sp$HBName,pos=4)

par(usr=c(0,1,0,1))
text(.05,0.95,"A",cex=2)

plot(fcpolysbi.sp,col='whitesmoke',border='grey80')

plot.nb(
 fcpolysbi.nb,
 coordinates(fcpolysbi.sp),
 col = 2,
 pch = 16,
 add=T
)

par(usr=c(0,1,0,1))
text(.05,0.95,"B",cex=2)

**References**

1. Statistics OfN. Postal Geography [cited 2016 Feb 28]. Available from: <http://webarchive.nationalarchives.gov.uk/20160105160709/http://www.ons.gov.uk/ons/guide-method/geography/beginner-s-guide/postal/index.html>

2. Baddeley A, Rubak E, Turner R. Spatial Point Patterns: Methodology and Applications with R. London: Chapman and Hall/CRC Press; 2016. 810 p.

3. Besag J. Discussion of Dr Ripley's paper. Journal of the Royal Statistical Society, . 1977;Series B(39):193-5.

4. Baddeley A, Diggle PJ, Hardegen A, Lawrence T, Milne RK, Nair G. On Tests of Spatial Pattern Based on Simulation Envelopes. Ecological Monographs 2014;84(3):477-89.

5. Scottish Government MS. Scotland’s national marine plan: consultation draft 2013. Available from: <http://www.nls.uk/scotgov/2013/9781782566519.pdf>.

6. Rey SJ, Anselin L. PySAL: A Python Library of Spatial Analytical Methods. Fischer MM, Getis A, editors. Berlin, Heidelberg: Springer Berlin Heidelberg2010.

7. Nelson JK, Brewer CA. Evaluating data stability in aggregation structures across spatial scales: revisiting the modifiable areal unit problem. Cartography and Geographic Information Science. 2017;44(1):35-50. doi: 10.1080/15230406.2015.1093431.
